# Identification and Verification of Biomarkers Related to Polyamine Metabolism in Diabetic Nephropathy

**DOI:** 10.1155/jdr/9539734

**Published:** 2025-12-30

**Authors:** Sen Zhou, Haiqian An, Hui Wei, Rong Wu, Zhe Wang, Minglong Liu, Tianxi Liu, Kan Li

**Affiliations:** ^1^ Department of Nephropathy, The First Hospital of Lanzhou University, Lanzhou, China, lzu.edu.cn; ^2^ The First Clinical Medical College, Lanzhou University, Lanzhou, China, lzu.edu.cn

**Keywords:** biomarkers, diabetic nephropathy, machine learning algorithm, polyamine metabolism, regulatory network

## Abstract

**Background:**

Kidney damage in chronic kidney disease patients is affected by the degradation products of polyamines. However, the effect of polyamine metabolism–related genes (PM‐RGs) in diabetic nephropathy (DN) is not clear. The objective of this study is to elucidate the potential correlation between PM‐RGs and DN.

**Methods:**

DN‐related datasets and 59 PM‐RGs were obtained from the public database. Then, Differentially Expressed Gene 1 (DEG 1) related to DN in GSE142153 and DEG 2 related to PM‐RGs were crossed to obtain intersection genes. The gene with the same expression trend in DEG 3 obtained in GSE185011 and DEG 1 was overlapped with the intersection gene to obtain the candidate genes. Thereafter, two machine learning algorithms and ROC curves were adopted to select biomarkers. Moreover, enrichment analysis, immune infiltration analysis, and drug prediction were implemented to further study the biomarkers. Finally, the expressions of biomarkers were analyzed in clinical samples assessed by RT‐qPCR and IHC.

**Results:**

KAZALD1, GLCE, and RPRD1B were identified as biomarkers for DN, with their area under the curve values being greater than 0.8. They were involved in multiple biological pathways, such as valine, leucine, and isoleucine degradation, cytokine–cytokine receptor interaction, and peroxisome. Furthermore, immune cells were found to correlate with biomarkers. For instance, the expression of KAZALD1 and RPRD1B showed positive correlations with naive CD8 T cells and M1 macrophages among other immune cells while exhibiting negative correlations with CD8 T cells, B cells, T helper cells, and others. Additionally, based on three biomarkers, 11 drugs (benzopyrene, Bisphenol A, ethinyl estradiol, etc.) were predicted. KAZALD1 and RPRD1B were notably highly expressed in clinical DN samples in RT‐qPCR and IHC.

**Conclusion:**

The research pinpointed KAZALD1, GLCE, and RPRD1B as biomarkers for DN, offering a novel target reference for diagnosing and treating DN.

## 1. Introduction

Diabetes has emerged as a major medical issue threatening human health in this century, with the prevalence of diabetes increasing significantly globally over the past few decades. By 2045, the number of individuals with diabetes is projected to rise to 700 million [1]. Diabetic nephropathy (DN) is a frequent and serious microvascular complication of diabetes, mainly marked by damage to the renal microvasculature and glomeruli, resulting in abnormal proteinuria and filtration. This condition may cause kidney failure in diabetic patients and is a leading cause of end‐stage renal disease (ESRD) globally [2]. Early DN often presents insidiously and progresses rapidly, with mild clinical symptoms that typically result in patients seeking medical attention only when the disease has reached Stage IV or beyond [3, 4]. Nevertheless, the development of DN is intricate and not fully comprehended, leading to less‐than‐ideal results. Furthermore, conventional therapeutic approaches that emphasize strict glycemic and blood pressure management are unable to halt the progression of DN to ESRD [5]. Therefore, an in‐depth study of the pathogenesis of DN and the identification of prospective therapeutic targets will provide an important basis for the development of innovative therapeutic strategies.

Polyamines, including spermidine (SPD), spermine, and putrescine, are important polycations naturally present in the biological world. They exist in mammalian cells at millimolar concentrations and play significant roles in the functions and replication of both normal and tumor cells [6]. Polyamines are vital for the normal growth of cells, and their depletion can lead to cytostasis [7]. Polyamine metabolism (PM) covers biosynthesis, catabolism, and transport and has a pervasive impact on cell proliferation, apoptosis, and gene regulation [8]. Studies have demonstrated that the breakdown products of polyamines can worsen kidney damage in individuals with chronic kidney disease, suggesting that polyamines might play a role in the development of kidney diseases [9]. It has also been shown that putrescine concentration is elevated in the plasma of patients diagnosed with DN, chronic glomerulonephritis, and nephrosclerosis, while the level of spermine is reduced [10]. A decrease in the polyamine metabolic pool, especially a drop in spermine levels, plays a crucial role in the advancement of DN [11]. Nevertheless, to further clarify the specific role of PM in DN, particularly their impact on renal pathological changes, more research is still required. Consequently, a thorough exploration of polyamine metabolism–associated genes (PM‐RGs) and their relationship with the initiation and progression of DN could provide valuable insights into the disease′s underlying mechanisms, potentially identifying new therapeutic targets and research directions for its treatment.

Currently, most research is focused on renal tissues in DN, with few studies investigating the pathogenesis of DN based on peripheral blood mononuclear cells (PBMCs). Therefore, this study utilizes the transcriptome dataset of PBMCs from DN patients to perform functional annotation, enrichment analysis, and interaction network construction of related genes. The aim is to identify potential biomarkers and key pathways associated with PM in DN, thereby providing a theoretical basis for the early diagnosis and precise treatment of the disease.

## 2. Materials and Methods

### 2.1. Data Extraction

DN‐related datasets (GSE142153 and GSE185011) were acquired from the Gene Expression Omnibus (GEO) database (https://www.ncbi.nlm.nih.gov/gds). GSE142153 dataset (GPL6480) consisted of 23 DN and 10 control PBMC samples [12]. GSE185011 dataset (GPL24676) included five DN and five control PBMC samples [13], which were utilized for expression verification. The 59 PM‐RGs in the REACTOME METABOLISM OF POLYAMINES gene set were acquired from the MSigDB (https://www.gsea-msigdb.org/gsea/msigdb/) by searching for the keyword “polyamine metabolism.”

### 2.2. Data Preprocessing

In this study, two independent datasets obtained from the GEO database were preprocessed as follows: For the microarray dataset GSE142153, the expression matrix that had been normalized through the platform′s standard procedure was extracted. The probe IDs were converted to official gene symbols using the GPL6480 annotation file, and for cases where multiple probes corresponded to the same gene, the probe with the highest average expression level was retained to eliminate redundancy. For the RNA‐seq dataset GSE185011, the raw data was provided in FPKM format (which had already been corrected for gene length and sequencing depth, so no additional normalization was required), and direct data cleaning and gene deduplication were performed. Both datasets were filtered to remove low‐expression genes according to the criterion of “retaining genes with an expression level > 0 in ≥ 50% of samples,” resulting in the final gene expression matrices for subsequent analysis.

### 2.3. *K*‐Means Clustering Algorithm and Differential Expression Analysis

The background gene set was derived from the GSE142153 dataset, which was intersected with 59 PM‐RGs to identify the intersection PM‐RGs. Then, all samples in GSE142153 were analyzed utilizing the cluster package (v 2.1.4) (https://www.bibsonomy.org/bibtex/2153b1c71326b16e5d93b998666537ee8/enitsirhc) to perform cluster analysis based on intersection PM‐RGs. Subsequently, principal component analysis (PCA) was employed to evaluate the degree of differentiation between the samples within the various clusters. Therewith, differential expression analysis conducted via the limma package (v 3.52.4) [14] was employed to identify Differentially Expressed Gene 1 (DEG 1) between the DN and control groups in the GSE142153 dataset and DEG 2 between different clusters. The resulting *p* values were adjusted for multiple testing using the Benjamini–Hochberg (BH) method to control the FDR. Genes with an FDR < 0.05 and |log ~ 2 ~ FC| > 0.5 were considered differentially expressed. The volcano map and heat map were generated to display the top 10 up‐ and downregulated DEGs applying ggplot2 (v 3.3.6) [15] and Circlize package (v 0.4.15) [16], respectively. Furthermore, to identify DEG 3 of DN and control samples in the GSE185011 dataset, differential expression analysis was processed via the limma package with the criteria of *p* < 0.05 and |log2FC| = 0.

### 2.4. Identification and Enrichment Analysis of Intersection Genes

The intersection of DEGs 1 and 2 yielded a set of genes that were differentially expressed in both datasets. In order to gain insight into the biological functions and pathways associated with the intersection genes, Gene Ontology (GO) and Kyoto Encyclopedia of Genes and Genomes (KEGG) enrichment analyses were conducted, and the resulting *p* values were adjusted for multiple comparisons using the BH method. An FDR < 0.05 was set as the threshold to identify significantly enriched terms. The interaction of intersection genes at the protein level was explored using the STRING database (http://www.string-db.org/) (interaction score of ≥ 0.7). Subsequently, the protein–protein interaction (PPI) network was visualized using Cytoscape software (v 3.10.1) [17].

### 2.5. Selection of Biomarkers

The upregulated genes in DEG 1 and DEG 3 and the downregulated genes in DEG 1 and DEG 3 were crossed with intersection genes, respectively, and then, candidate genes were obtained by their union. Subsequently, two machine learning algorithms, the LASSO and Boruta, were separately utilized to select feature genes through glmnet (v 4.1‐8) [18] and the Boruta package (v 8.0.0) [19]. Specifically, LASSO generated a lambda minimum model to select feature genes, whose coefficient was not penalized as 0. Boruta conducted multiple iterations to evaluate gene importance (*Z*‐score) and selected feature genes within the most stable outcomes. Candidate biomarkers were obtained by taking the intersection of feature genes derived from these two machine learning algorithms. The ability of candidate biomarkers to distinguish between DN and control samples was assessed using the receiver operating characteristic (ROC) curve. Biomarkers were identified as those with an area under the curve (AUC) exceeding 0.7.

### 2.6. Enrichment Analysis of Biomarkers

Gene set enrichment analysis (GSEA) was executed using the GSEA function with the background gene set c2.cp.kegg.v2023.1.Hs.symbols.gmt obtained via the msigdbr package (v 7.5.1) [20] to identify biological functions associated with biomarkers. In detail, Spearman′s correlation analysis between biomarkers and all genes in the GSE142153 dataset was processed by the psych package (v 2.4.1) [21], and their correlation coefficients were ranked for GSEA (adj.*p* < 0.05). In addition, SPEED2 (https://speed2.sys-bio.net/) was utilized to explore the activity of upstream pathways of biomarkers. The grade change of upstream pathways was tested and quantified by Bates.

### 2.7. Immune Infiltration Analysis

To evaluate immune infiltration levels in the DN and control groups from the GSE142153 dataset, the xCell algorithm was used to measure the abundance of 64 types of immune cells in the samples. The Wilcoxon test (*p* < 0.05) was used to compare the abundance of immune infiltration between the DN and control groups. A boxplot of the results was drafted by the ggplot2 package. Furthermore, the Spearman correlation of immune cells with biomarkers was analyzed by the pheatmap package (v 1.0.12) [22](*p* < 0.05).

### 2.8. Creation of Regulatory Network and Drug Prediction

To identify potential regulatory mechanisms, microRNA (miRNA)‐targeting biomarkers were predicted by the miRDB (http://mirdb.org) database. Furthermore, the top 10 miRNAs, ranked by their score in miRDB, targeting each biomarker′s long noncoding RNA (lncRNAs) were predicted in the starBase database (http://starbase.sysu.edu.cn/). Afterwards, based on the top 10 miRNAs, lncRNAs, and biomarkers, a lncRNA–miRNA–biomarker regulatory network was constructed. The drugs targeting biomarkers were predicted in the Comparative Toxicogenomics Database (CTD) (http://ctdbase.org/).

### 2.9. Reverse Transcription Quantitative Polymerase Chain Reaction (RT‐qPCR)

The expression of biomarkers was analyzed in GSE142153 and GSE185011, as well as in clinical samples assessed by RT‐qPCR. PBMC samples were acquired from The First Hospital of Lanzhou University, consisting of five control specimens and five DN specimens. Informed consent forms were completed and signed by all participants, while the ethical approval was granted by the Ethics Committee of The First Hospital of Lanzhou University (LDYYLL‐2024‐525). The samples underwent RNA extraction and purification using TRIzol (Ambion, Austin, United States). Subsequently, cDNA synthesis was performed on the extracted RNA using the SureScript First‐Strand cDNA Synthesis Kit (ServiceBio, Wuhan, China). The resulting cDNA was analyzed using 2xUniversal Blue SYBR Green qPCR Master Mix (ServiceBio) along with specific primer sequences (Table S1). RT‐qPCR amplification was carried out under a thermal cycling protocol. Data analysis utilized the 2^−*Δ* *Δ*Ct^ method with GAPDH serving as the internal reference gene for normalization.

### 2.10. Immunohistochemistry (IHC)

According to the Ethics Committee–approved protocol, paraffin‐embedded kidney tissue sections were obtained from The First Hospital of Lanzhou University, including samples from DN tissue (*n* = 5) and paracancerous tissue (*n* = 5), with informed consent obtained from all patients. Immunohistochemical processing was executed to detect GLCE (1:200, Bioss, China), KAZALD1 (1:200, Bioss, China), and RPRD1B (1:200, PTGLAB, United States). The sections were deparaffinized using xylene, rehydrated with a series of ethanol concentrations, and then rinsed with distilled water. Antigen retrieval was performed, and endogenous peroxidase activity was blocked using 3% hydrogen peroxide for 25 min. Following a 3% BSA block, the primary antibodies were left to incubate overnight at 4°C. Following PBS washes, HRP‐conjugated secondary antibodies were applied for 50 min. DAB solution was used for color development, and hematoxylin was applied for counterstaining the nuclei. The sections were then dehydrated, cleared with xylene, mounted using neutral resin, and examined under a Nikon E100 light microscope.

### 2.11. Statistical Analysis

The R software (v 4.2.3) was used to process and analyze the data. The *p* value < 0.05 was considered statistically significant.

## 3. Results

### 3.1. Intersection Genes Were Associated With Apoptosis

The gene set of the GSE142153 dataset and 59 PM‐RGs exhibited a total of 56 intersection PM‐RGs, based on which all samples were successfully classified into two distinct clusters. PCA results showed that two clusters of the sample were well distinguished (Figure 1a). Through differential expression analysis, 1445 DEG 1, including 707 upregulated and 738 downregulated DEGs, were identified between the DN and control groups in the GSE142153 dataset (FDR < 0.05) (Figure 1b,c). Besides, 1812 DEG 2 (1202 upregulated and 610 downregulated) were obtained between Cluster 1 and Cluster 2 (FDR < 0.05) (Figure 1d,e). By taking the intersection of 1445 DEG 1 and 1812 DEG 2, 524 intersection genes were acquired (Figure 1f). Enrichment analysis showed that 31 GO terms and seven KEGG pathways were enriched by intersection genes. GO terms included positive regulation of cytokine production and oxygen transport (FDR < 0.05) (Figure 1g). KEGG pathways consisted of lipid and atherosclerosis, the MAPK signaling pathway, and apoptosis (FDR < 0.05) (Figure 1h). It is worth noting that PM is widely involved in apoptosis, consistent with our results. Moreover, a PPI network was constructed with 181 nodes and 194 edges (Figure S1). Notably, IL‐1B and IL‐6 had the most correlation with other genes.

Figure 1Intersection genes were associated with apoptosis. (a) *K*‐means clustering based on 56 PM‐RGs in the training set GSE142153. (b) Volcano plot of DEG 1 in the disease and normal groups. Red dots indicate upregulated differential expression, green dots indicate downregulated differential expression, and grey dots indicate no significant difference in these genes. (c) Heat map of DEG 1 between disease and normal groups. Low expression in green, high expression in red. (d) Volcano map of DEG 2 between Cluster 1 and Cluster 2. Red dots indicate upregulated differential expression, green dots indicate downregulated differential expression, and grey dots indicate no significant difference in these genes. (e) Heatmap of DEG 2 between Cluster 1 and Cluster 2. Low expression in green, high expression in red. (f) Venn diagram for the intersection of DEG 1 and DEG 2. (g) GO enrichment analysis of intersection genes. (h) KEGG enrichment analysis of intersection genes.

**Figure:**
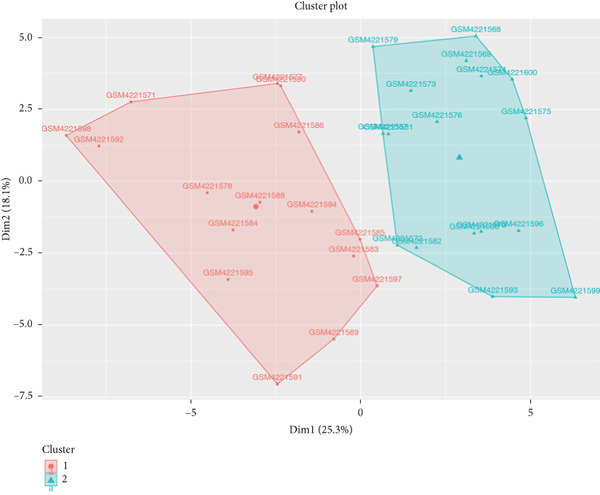
(a)

**Figure:**
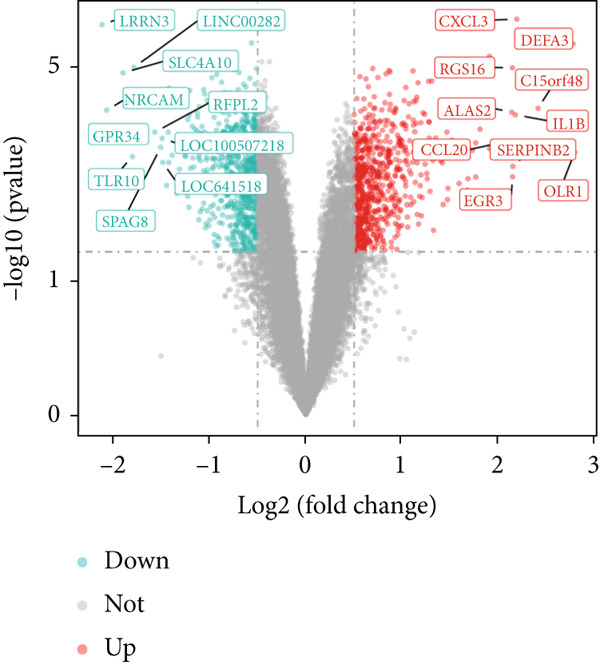
(b)

**Figure:**
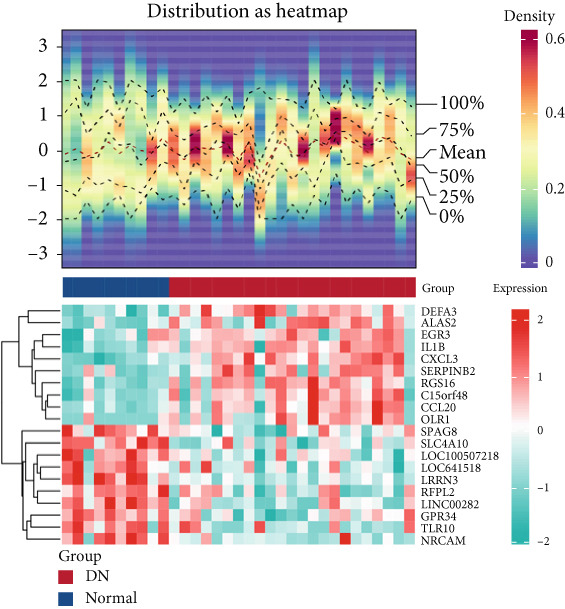
(c)

**Figure:**
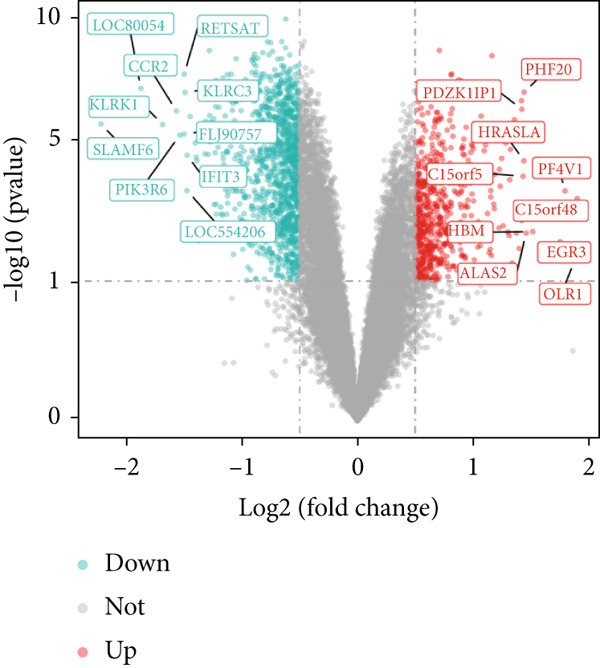
(d)

**Figure:**
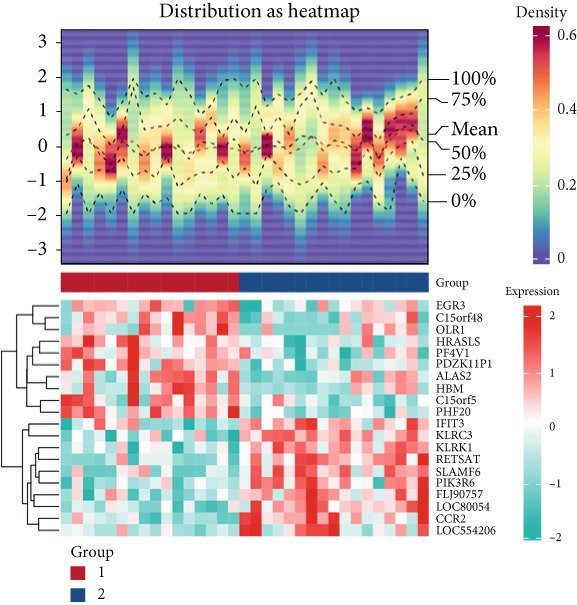
(e)

**Figure:**
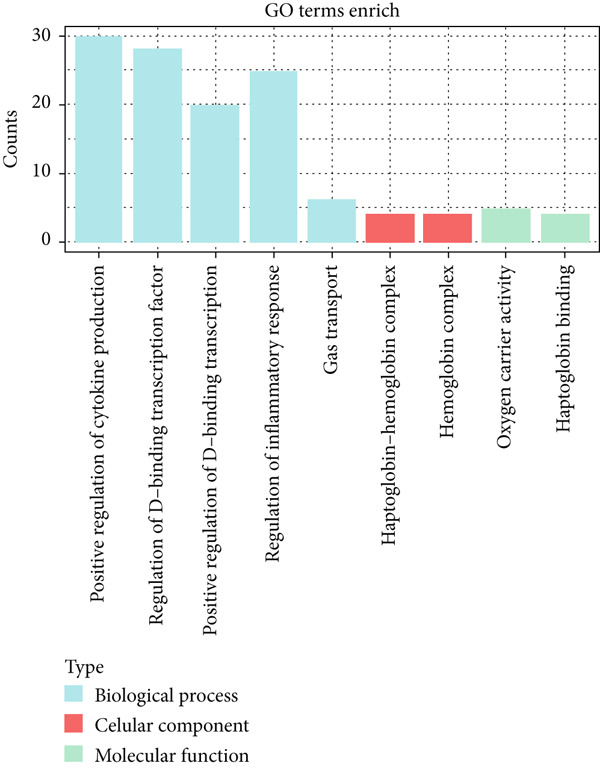
(f)

**Figure:**
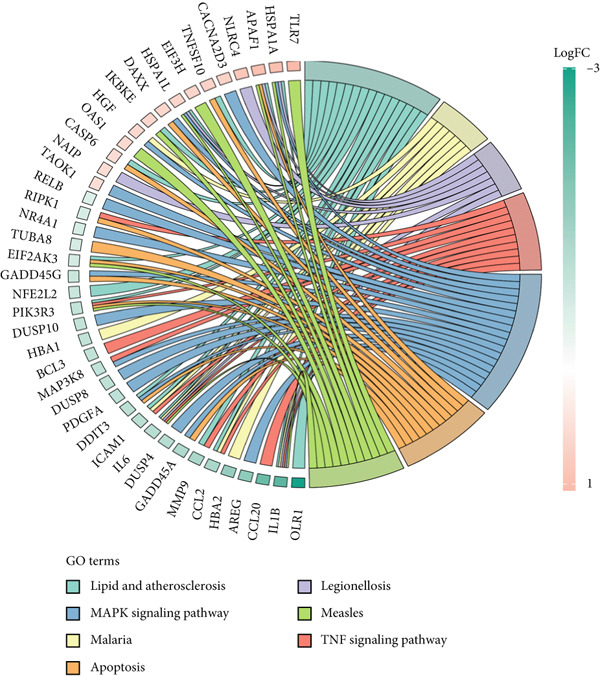
(g)

**Figure:**
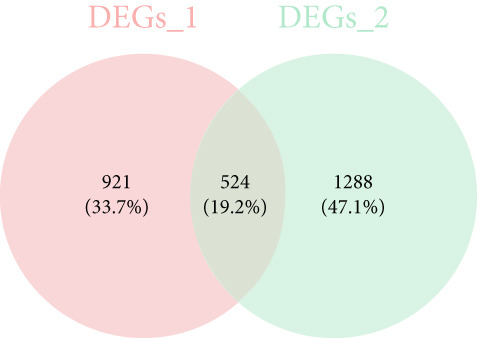
(h)

### 3.2. KAZALD1, GLCE, and RPRD1B Were Identified as Biomarkers for DN

Totally 1593 DEG 3 (1179 upregulated and 414 downregulated) were selected between the DN and control groups in the GSE185011 dataset (*p* < 0.05). The co‐upregulated and co‐downregulated genes in DEG 1 and DEG 3 were crossed with intersection genes, respectively, and then, eight candidate genes were obtained by their union (Figure 2a,b). The LASSO model with lambda.min equal to 0.076 included three feature genes whose coefficients were not penalized as 0, which were KAZALD1, GLCE, and RPRD1B (Figure 2c,d). Boruta identified five feature genes (KAZALD1, GLCE, RPRD1B, ZNF250, and MED21) (Figure 2e). KAZALD1, GLCE, and RPRD1B were commonly identified by LASSO and Boruta; thus, they were candidate biomarkers (Figure 2f). Moreover, the AUC values of these three candidate biomarkers exceed 0.8 in both the GSE142153 and GSE185011 datasets, indicating their robust diagnostic capability for DN and identifying them as biomarkers (Figure 2g,h).

Figure 2
*KAZALD1*, *GLCE*, and *RPRD1B* were identified as biomarkers for DN. (a, b) Venn plots of co‐upregulated and co‐downregulated genes in DEG 1 and DEG 3 crossed with intersection genes, respectively. (c, d) LASSO regression screening of feature genes. (e) Boruta′s algorithm screens for feature genes. (f) Venn diagram of the intersection for feature genes obtained by LASSO and Boruta. (g, h) ROC curves for the three candidate biomarkers in the GSE142153 and GSE185011 datasets.

**Figure:**
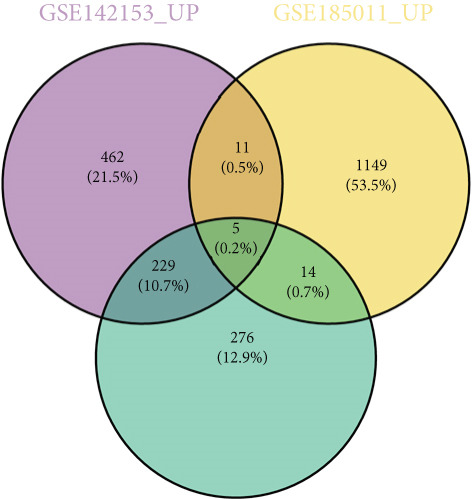
(a)

**Figure:**
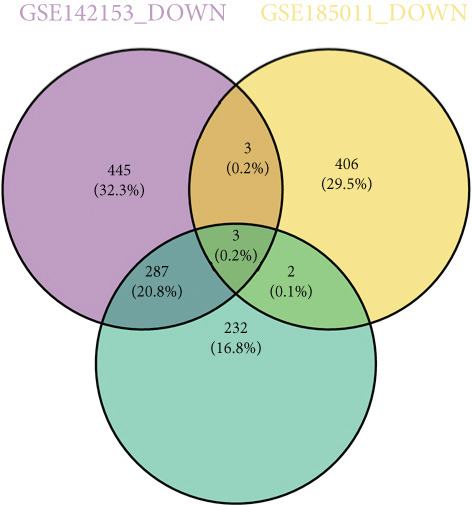
(b)

**Figure:**
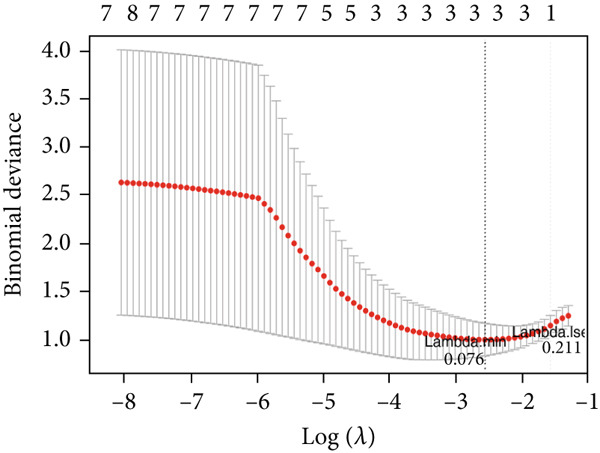
(c)

**Figure:**
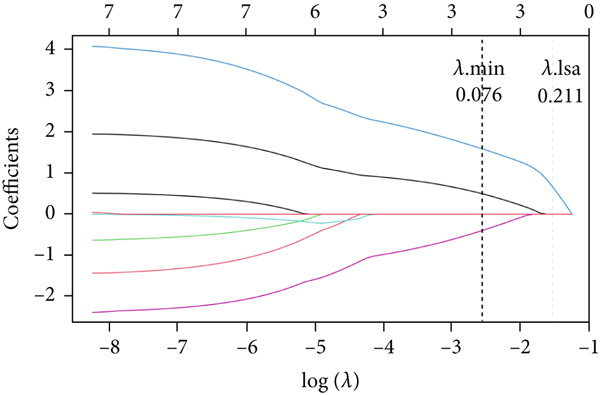
(d)

**Figure:**
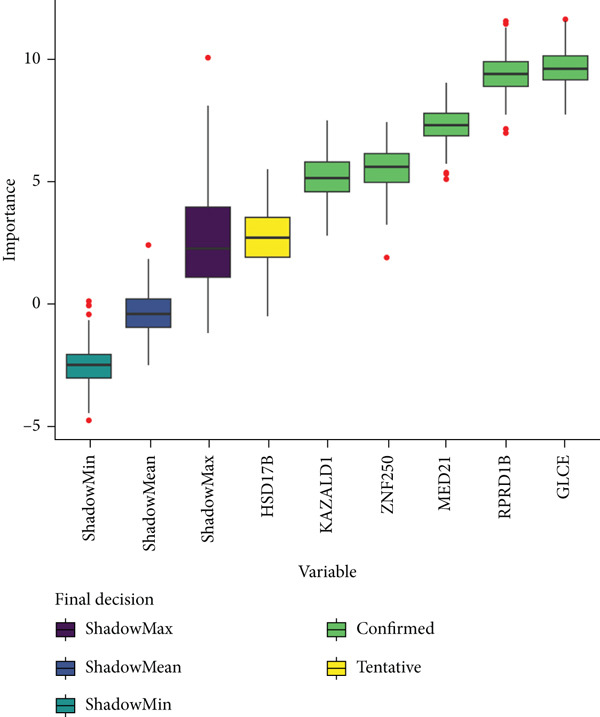
(e)

**Figure:**
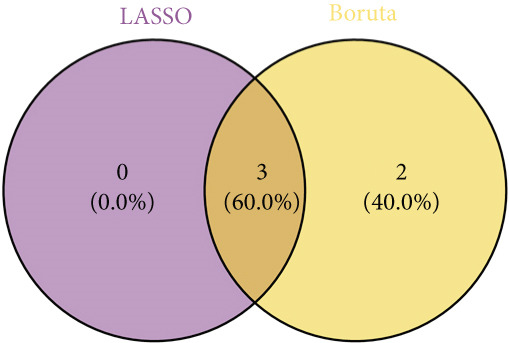
(f)

**Figure:**
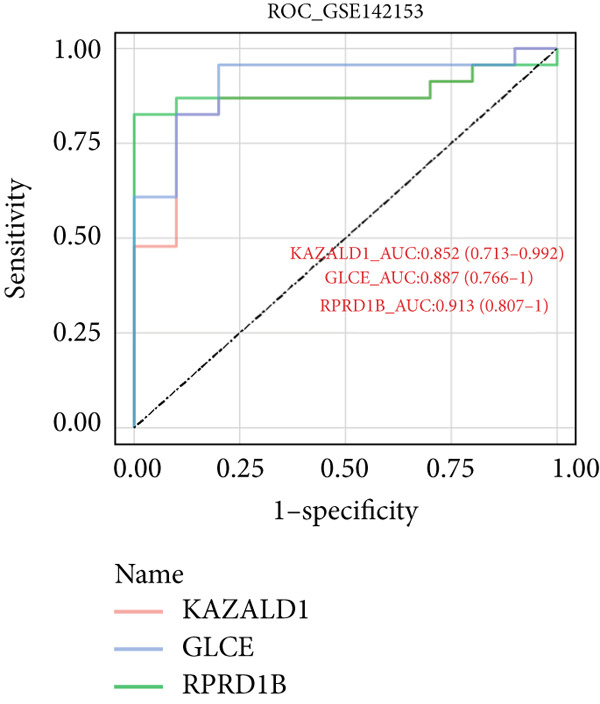
(g)

**Figure:**
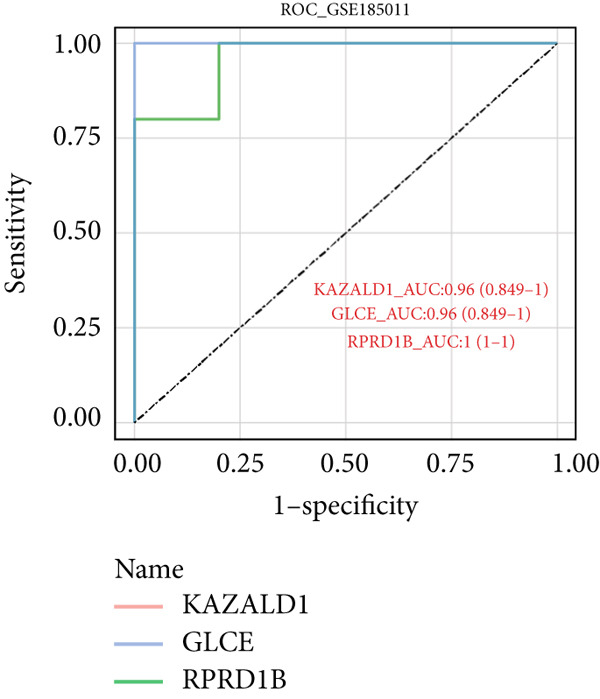
(h)

### 3.3. Biomarkers Were Involved in Multiple Biological Pathways

In the GSE142153 dataset, GSEA was conducted to investigate biological functions and pathways involved in biomarkers. All biomarkers were significantly associated with valine, leucine, and isoleucine degradation, cytokine–cytokine receptor interaction, and peroxisome‐related pathways (Figures 3a, 3b, and 3c, Table S2). Moreover, the expression of biomarkers was upregulated in the upstream active pathways, including hypoxia, notch, and p53, and was downregulated in the estrogen, TGF‐*β*, and other pathways (Figure 3d). Particularly, the downward trends of biomarkers in estrogen were significant.

Figure 3Biomarkers were involved in multiple biological pathways. (a–c) GSEA results for biomarkers (*KAZALD1*, *GLCE*, and *RPRD1B*). (d) SPEED2 signaling pathway enrichment analysis of biomarkers.

**Figure:**
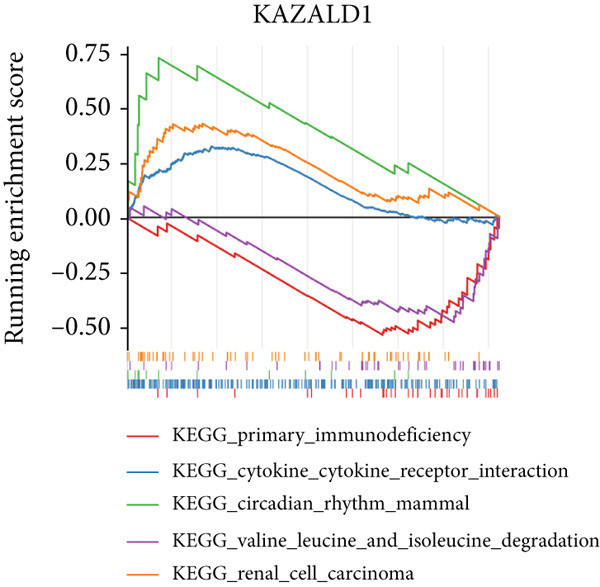
(a)

**Figure:**
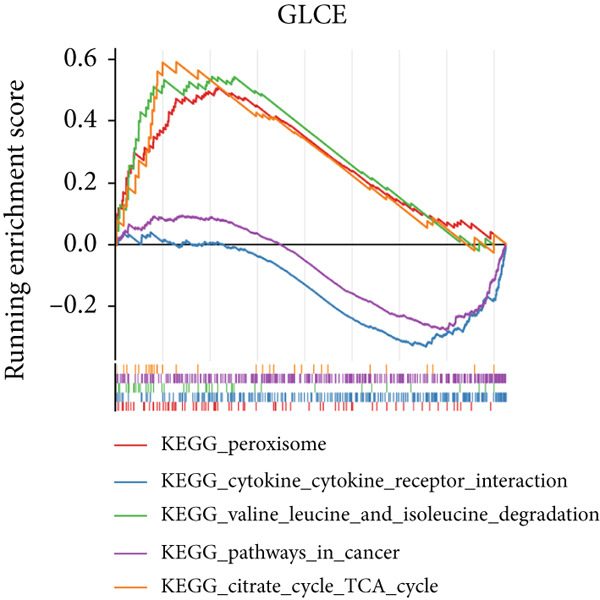
(b)

**Figure:**
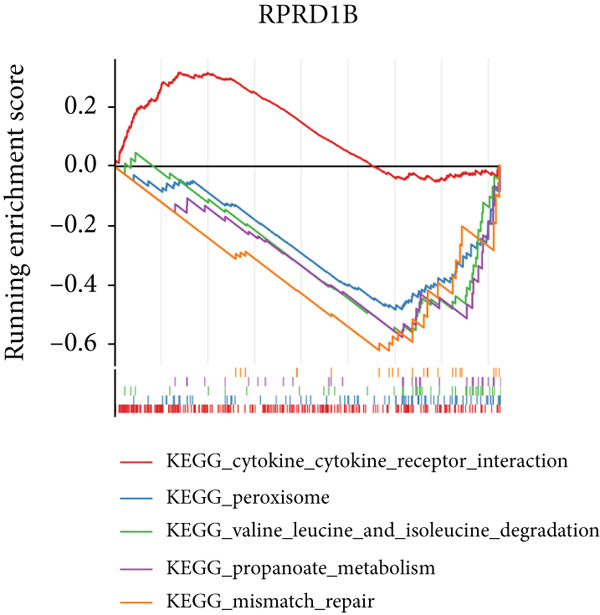
(c)

**Figure:**
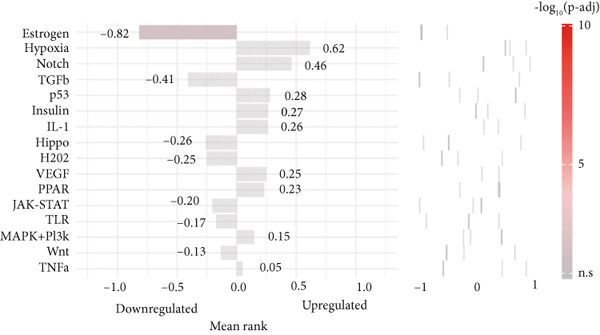
(d)

### 3.4. Immune Cells Existed in Correlation With Biomarkers

The distribution of 64 immune cells in DN and control samples from the GSE142153 dataset is depicted in Figure 4a. Out of these, there were notable variations in infiltration between the two groups for 19 immune cell types, including adipocytes, B cells, and naive CD4 T cells (*p* < 0.05) (Figure 4b). Importantly, the expression of KAZALD1 and RPRD1B exhibited positive correlations with naïve CD8 T cells, M1 macrophages, and other immune cells (*r* > 0, *p* < 0.05) while showing a negative correlation with CD8 T cells, B cells, and so on (*r* < 0, *p* < 0.05). In contrast, the relationship between GLCE and immune cells displayed an opposite trend compared to the other two biomarkers (Figure 4c).

Figure 4Immune cells showed a correlation with biomarkers. (a) Stacked plot of immune cell abundance. (b) Differential expression of immune cells. ns indicates *p* > 0.05,  ^∗^ *p* < 0.05,  ^∗∗^ *p* < 0.01,  ^∗∗∗^ *p* < 0.001, and  ^∗∗∗∗^ *p* < 0.0001. (c) Correlation analysis between biomarkers and differential immune cells.

**Figure:**
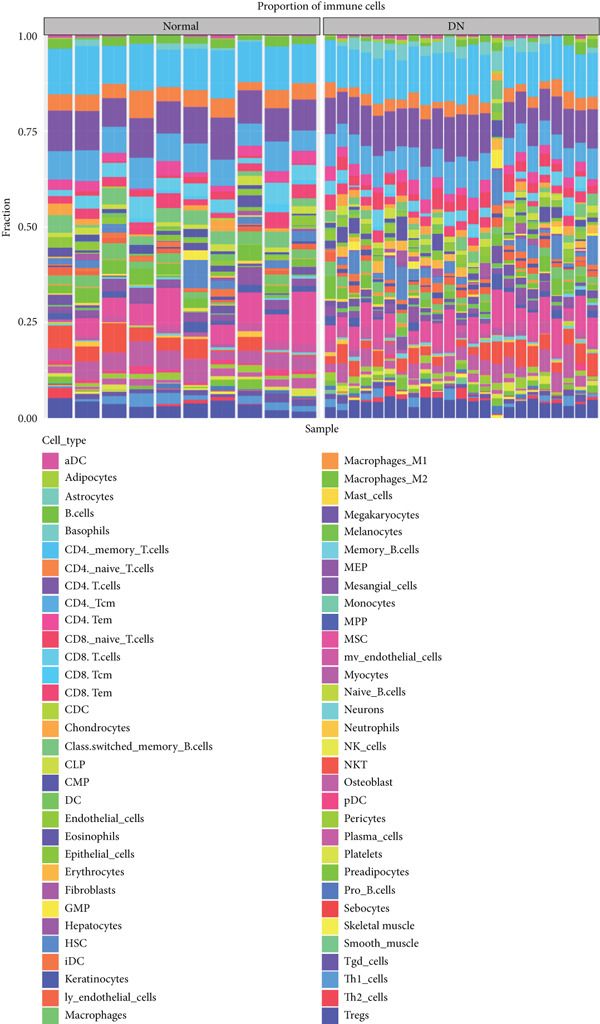
(a)

**Figure:**
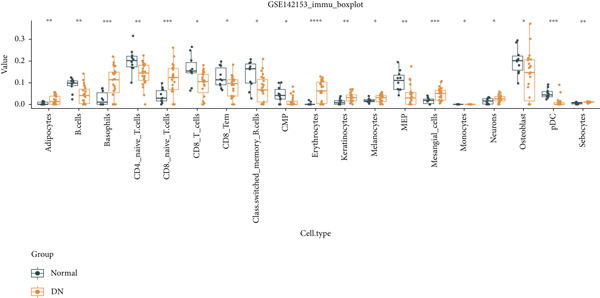
(b)

**Figure:**
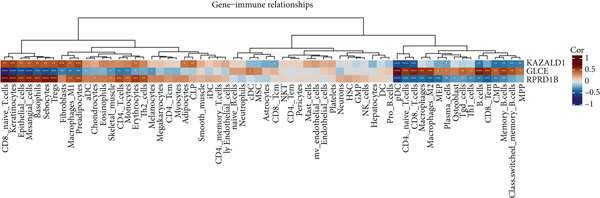
(c)

### 3.5. Biomarkers Were Regulated by Multiple Molecules Simultaneously

A lncRNA–miRNA–biomarker regulatory network was constructed, consisting of three biomarkers, 30 miRNAs, and 27 lncRNAs (Figure 5a, Table S3). The regulation of biomarkers involved the simultaneous action of multiple molecules. For example, the collaborative action of SNHG12, NEAT1, LINC02595, SNHG16, and AL109615.3 was involved in the regulation of GLCE through hsa‐miR‐218‐5p. Based on three biomarkers, 11 drugs (benzopyrene, Bisphenol A, ethinyl estradiol, etc.) were predicted (Figure 5b). Notably, all three biomarkers were targeted by Bisphenol A, and KAZALD1 and GLCE were cotargeted by benzopyrene and ethinyl estradiol. These drugs played a certain role in diseases and might become potential drugs for DN.

Figure 5Biomarkers were regulated by multiple molecules simultaneously. (a) Molecular regulatory networks of biomarkers. Biomarkers are in red, miRNAs in green, and lncRNAs in blue, with the connecting lines indicating that they are related. (b) Drug prediction networks for biomarkers. Biomarkers are in red, drugs are in green, and the connecting line indicates that they are related.

**Figure:**
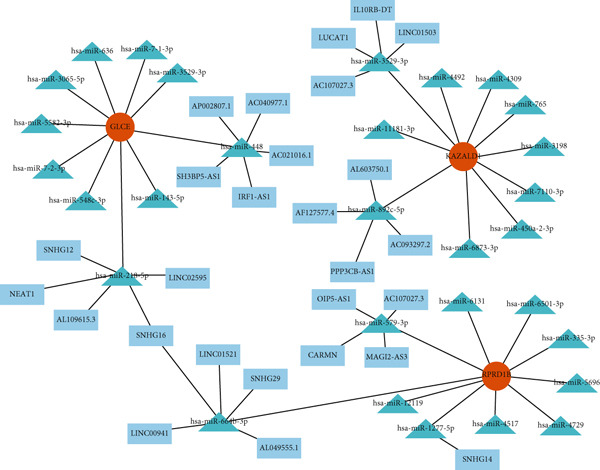
(a)

**Figure:**
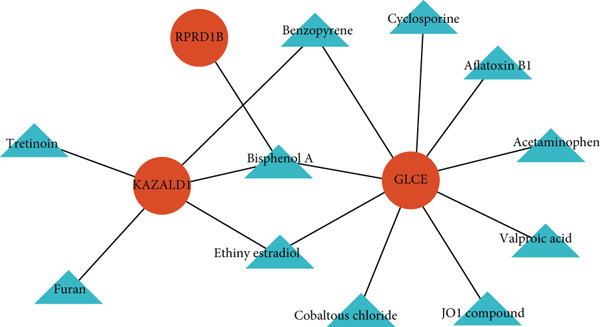
(b)

### 3.6. Validation of Biomarker Expression

The expression levels of KAZALD1 and RPRD1B were memorably upregulated in the DN group, whereas GLCE exhibited notable downregulation in the DN group in both GSE142153 and GSE185011 (Figure 6a,b). To further validate biomarker expression, sample quality and availability were assessed using HE and PAS staining (Figure 6c). Subsequent validation through PCR and IHC demonstrated that KAZALD1 and RPRD1B were significantly overexpressed in clinical DN samples, aligning with the dataset results. However, the expression pattern of GLCE did not correspond to the observed trends in the dataset (Figure 6d,e).

Figure 6Validation of biomarker expression. (a) The expression level analysis of biomarkers in GSE142153. (b) The expression level analysis of biomarkers in GSE185011. (c) Sample quality and usability were assessed using HE and PAS stains. (d, e) The expression level analysis of biomarkers in clinical DN samples.

**Figure:**
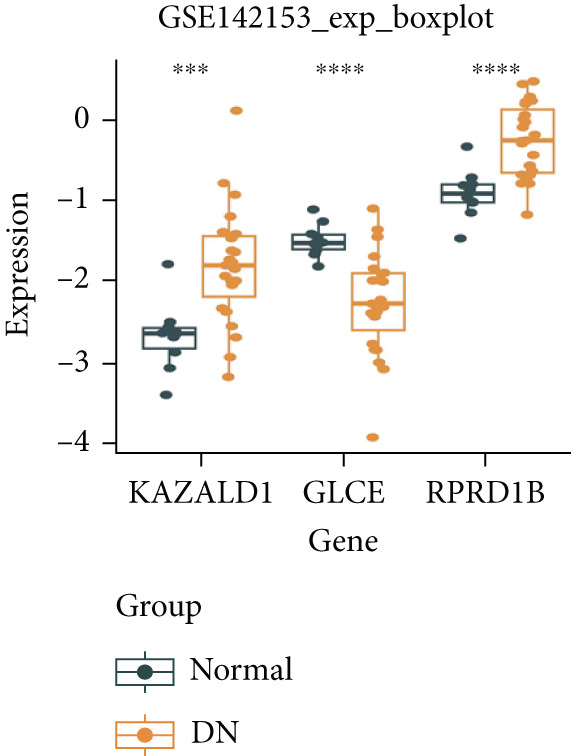
(a)

**Figure:**
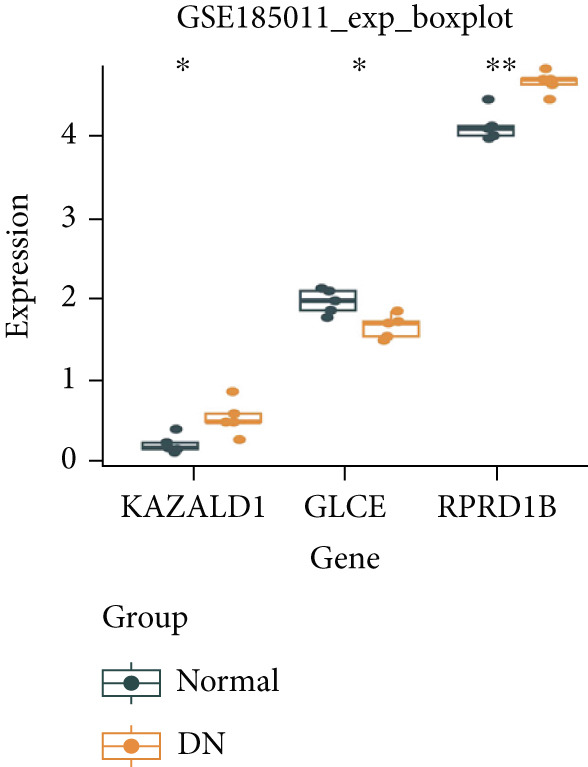
(b)

**Figure:**
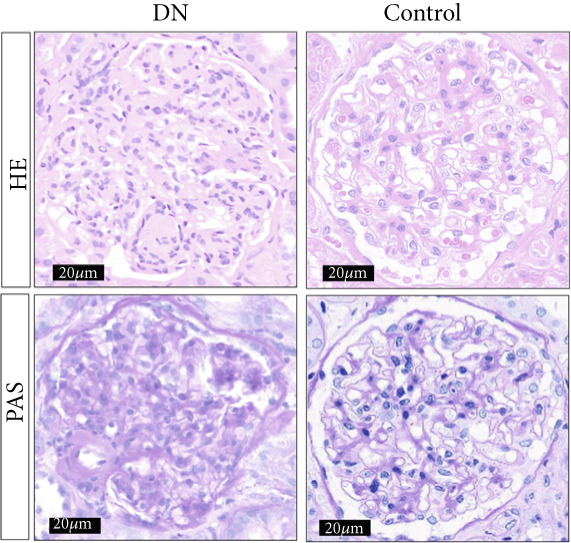
(c)

**Figure:**
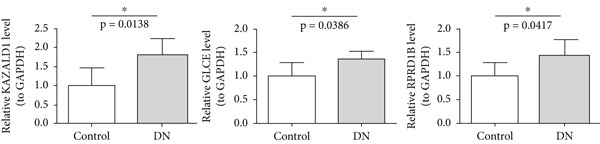
(d)

**Figure:**
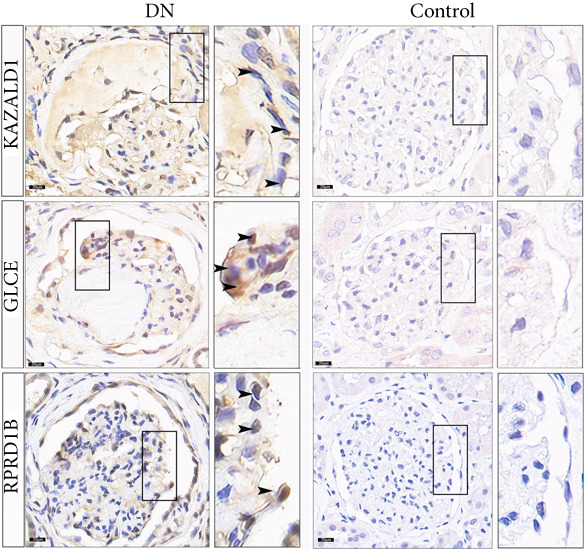
(e)

## 4. Discussion

DN is a prevalent and deadly complication in individuals with diabetes, and it is a primary cause of ESRD [23]. Its pathogenesis involves dysregulation of complex metabolic pathways, resulting in progressive damage to renal structure and function [24]. Recently, studies have increasingly concentrated on the involvement of the PM in the development of DN. Polyamines are small, positively charged molecules that regulate cell growth, differentiation, and gene expression [25]. Disruption of PM is closely associated with the development and progression of chronic kidney disease. Research indicates that hyperglycemia can cause abnormal PM in rat kidney tissues, triggering podocyte apoptosis and inhibiting autophagy, which may contribute significantly to renal damage in DN [11]. Modulating PM might help mitigate renal injury and improve the prognosis of DN [11]. Therefore, an in‐depth investigation into the role of PM‐RGs in DN could uncover their specific mechanisms in disease progression, thereby providing a theoretical basis for developing new therapeutic targets and strategies. Through PPI analysis, we identified extensive interactions between TGF‐*β*, IL‐6, and other PM‐RGs. This suggests their potential roles in DN, possibly contributing to the fibrosis process by interacting with proteins associated with the TGF‐*β* signaling pathway.

The present study employed differential gene expression analysis, machine learning, and ROC curves to identify three biomarkers associated with PM and DN: KAZALD1, GLCE, and RPRD1B. Differential expression of these markers may provide new clues for early diagnosis and intervention in DN. KAZALD1 is a member of the insulin growth factor–binding protein superfamily [26] and is crucial in numerous physiological and pathological processes. Research indicates that the hypomethylation of the KAZALD1 promoter serves as a crucial prognostic marker for glioblastoma [26]. Recent studies indicate that KAZALD1 could be a promising biomarker for the early identification of renal ischemia–reperfusion injury (IRI) [27]. Previous studies have implicated KAZALD1 in genitourinary abnormalities [28]. Notably, this study reveals for the first time that PM is associated with varying expression levels of KAZALD1 in DN patients. GLCE plays a crucial role in the production of heparan sulfate (HS) by transforming D‐glucuronic acid into L‐iduronic acid, thereby enhancing HS activity [29]. The literature suggests that HS is reduced in the glomerular basement membrane of DN patients and that the reduction in HS is inversely correlated with proteinuria [30]. In addition, GLCE deficiency in the liver promotes obesity, and reduced GLCE expression further reduces hepatic secretion of GDF15, which in turn interferes with lipid metabolism and energy homeostasis [31]. Thus, it is hypothesized that the GLCE may participate in DN through its influence on HS metabolism, likely exhibiting a positive role. RPRD1B is alternatively referred to as CREPT, a protein associated with the cell cycle and increased expression in tumors [32]. It contains a C‐terminal domain–interacting domain [32]. Multiple studies indicate that RPRD1B is involved in regulating the transcription of Cyclin D1 [33]. Moreover, the upregulation of Cyclin D1 can promote the proliferation of renal mesangial cells [34–36], contributing to renal injury. Based on the findings, RPRD1B may be involved in renal damage by affecting the expression of Cyclin D1. In our RT‐qPCR and IHC results, significant differences in the expression levels of KAZALD1, RPRD1B, and GLCE were also observed, confirming the diagnostic value of these biomarkers in DN. In this study, discrepancies were observed between bioinformatic predictions based on PBMCs and immunohistochemical validation results from renal tissues regarding GLCE expression. These differences may be attributed to several factors. From a tissue‐specific perspective, GLCE, as a key enzyme in the synthesis of sulfated HS [37], exhibits differential expression patterns across various tissues. In PBMCs, its expression may reflect systemic metabolic status, whereas in renal tissue, GLCE is directly involved in maintaining the structural integrity and function of the glomerular basement membrane [38, 39]. This tissue distribution disparity likely underlies the inconsistent findings. From the viewpoint of disease heterogeneity, DN itself is characterized by considerable heterogeneity [40, 41]. Variations in disease stage, pathological classification, and comorbidities among patients can lead to fluctuations in GLCE expression within the local renal microenvironment, further complicating result interpretation. These findings suggest that GLCE may play a dual role in DN, participating both in early systemic metabolic responses and exhibiting dynamic changes during local lesion progression. To elucidate its precise mechanisms, future studies should focus on longitudinal monitoring of GLCE expression profiles in peripheral blood and renal tissues to systematically dissect its tissue‐ and stage‐specific functions in DN.

Although this study identified KAZALD1, GLCE, and RPRD1B as biomarkers associated with PM and demonstrated significant expression changes in DN, the direct mechanistic links between these biomarkers and the core biochemical pathways of PM require further validation. PM deeply participates in the pathological progression of DN by regulating key biological processes such as cell proliferation, apoptosis, autophagy, and oxidative stress [42, 43]. Based on existing literature, we hypothesize that these three biomarkers may interact with the polyamine metabolic network through the following mechanisms: Specifically, KAZALD1, a member of the IGFBP superfamily [44], may have its expression finely regulated by polyamine levels, as polyamines have been established as critical modulators of multiple growth factor signaling pathways [45, 46]. GLCE catalyzes the synthesis of sulfated HS chains, which can specifically bind polyamines and thereby influence their distribution and functional activity within the cellular microenvironment [37]. This suggests that GLCE may indirectly regulate extracellular matrix retention and signal transduction of polyamines by modulating HS structure. RPRD1B, an important cell cycle regulatory factor [47], may be functionally coregulated with PM; together, they potentially form a functional synergy driving key pathological processes in DN, such as glomerular mesangial cell proliferation. Collectively, these mechanistic hypotheses outline a potential interaction network between PM and biomarker expression, providing a novel theoretical framework for understanding DN pathogenesis.

The results from GSEA revealed that these biomarkers are closely associated with pathways involving valine, leucine, and isoleucine degradation, cytokine–cytokine receptor interactions, and peroxisome functions. This suggests that PM may be crucial in the development and progression of DN. Branched‐chain amino acids (BCAAs) (valine, leucine, and isoleucine) are among the most abundant essential amino acids in the human body and serve as key substrates for the synthesis of nitrogen‐containing compounds. They additionally control glucose metabolism via the PI3K/AKT/mTOR signaling pathway [48]. As DN advances, there is a notable decrease in serum BCAA levels, which are independently linked to indicators of kidney function [49]. This study further revealed that the pathway of valine, leucine, and isoleucine degradation is more active in the DN group, indicating that PM could influence the occurrence of DN through its involvement in BCAA degradation. The role of the cytokine–cytokine receptor interaction pathway in the pathophysiology of DN has also been documented [13, 50]. Moreover, peroxisome dysfunction can worsen kidney damage by decreasing oxidative stress and apoptosis caused by high blood sugar while enhancing endothelial dysfunction, thus aiding in the relief of kidney injury in DN [51, 52]. As shown in Figure 3d, SPEED2 analysis revealed that KAZALD1 and RPRD1B were upregulated in hypoxia and p53 pathways, suggesting their potential role in oxidative stress response. In conclusion, the pathways enriched in this study, including cytokine–cytokine receptor interaction and peroxisome function, are involved in the disease process of DN and provide a theoretical basis for further understanding of the pathological mechanism of DN.

This study also investigated the correlation between biomarkers and differentially expressed immune cells. Overall, KAZALD1 and RPRD1B showed a significantly positive correlation with naïve CD8 T cells and M1 macrophages, while they showed a negative correlation with CD8 T cells, B cells, and TH1 cells. GLCE showed an opposite correlation trend with immune cells compared to the other two biomarkers. Inflammation and immune responses play critical roles in the progression of DN [53, 54]. Numerous studies have indicated that the aberrant activation of CD8+ T cells and B cells is important in the development of DN [55–57]. A biogenic polyamine, SPD, can enhance the activity of fatty acid oxidation (FAO), mitochondrial activity, and cytotoxic function of CD8+ T cells, with this mechanism being more pronounced in naïve CD8+ T cells [58]. Conversely, spermine can competitively inhibit the FAO activity of SPD [58], suggesting that PM may contribute to the progression of DN by modulating the activation and function of CD8+ T cells. M1 macrophages are a subtype of macrophages characterized by proinflammatory properties, and in DN, an increase in M1 macrophage polarization promotes the secretion of several proinflammatory cytokines, exacerbating renal inflammation and contributing to renal injury and fibrosis [54, 59, 60]. Importantly, polyamines are involved in regulating M1 macrophage function and polarization, playing a negative regulatory role in the proinflammatory response of M1 macrophages [61]. This hints at the possibility of inhibiting the proinflammatory response of M1 macrophages by modulating polyamine metabolic pathways, thus improving the progression of DN. TH1 cells are crucial in inducing renal fibrosis, contributing to the onset and progression of DN [62, 63]. Polyamines can influence the function and activity of TH1 cells, while TH1 cells can also regulate the synthesis and metabolism of polyamines. This interaction is crucial for maintaining immune balance and resisting pathogenic infections [54, 59]. These findings not only provide new insights into the immunoregulatory mechanisms of DN but also offer possible targets for future immunotherapy‐based clinical interventions.

In addition, we predicted drugs associated with the identified biomarkers, including compounds such as Bisphenol A, ethinyl estradiol, and benzopyrene, which exhibit various potential mechanisms of action. Bisphenol A, an endocrine disruptor, has been shown to affect insulin sensitivity and glucose metabolism, potentially exacerbating diabetes‐related kidney damage [64]. Its mechanisms may involve promoting renal fibrosis through oxidative stress and inflammatory pathways and further aggravating DN progression by influencing PM‐RGs such as KAZALD1, GLCE, and RPRD1B. However, due to its toxicity and widespread presence in the environment, the clinical application of Bisphenol A warrants cautious evaluation. Ethinyl estradiol, a synthetic estrogen commonly used in hormone therapy, has been reported to exert anti‐inflammatory and antifibrotic effects by modulating the TGF‐*β* signaling pathway [65]. Additionally, ethinyl estradiol may improve DN progression by regulating the expression of PM‐RGs. Currently, direct evidence regarding these drugs′ specific mechanisms in DN is limited, highlighting the need for additional experimental validation.

In conclusion, this study employed bioinformatics approaches to investigate the relationship between DN and PM using transcriptomic data and identified three biomarkers associated with both DN and PM, which were validated in clinical samples. Through a comprehensive analysis of gene functions, pathway enrichment, and network regulation, we found that PM may play a role in the onset and progression of DN through various biological processes. These three genes could serve as promising targets for the treatment of DN and offer new insights into therapeutic strategies for DN patients. Additionally, this study explored potential drug targets, providing valuable information for the development of novel therapeutic approaches for DN. These results enhance our comprehension of DN pathogenesis and establish a foundation for future clinical studies and treatments, offering substantial scientific and clinical importance.

However, this study has certain limitations. First, analyses based on public databases are constrained by limited sample sizes, and the generalizability of the results requires further validation. Second, potential batch effects exist among different datasets. Moreover, although retrospective data analysis revealed associations between gene expression and disease, causal relationships remain unconfirmed. Additionally, functional experimental validation of the candidate biomarkers is lacking, and the inconsistency in GLCE expression between clinical samples and bioinformatic analyses remains unresolved. Future studies should validate the value of these biomarkers in larger scale, multicenter cohorts and prioritize the use of standardized detection techniques such as RNA‐seq to fundamentally eliminate interplatform batch effects. Simultaneously, in‐depth cellular and animal experiments are needed to elucidate the specific mechanisms by which these biomarkers regulate apoptosis, fibrosis, and inflammation. Integrating multiomics data will facilitate the construction of a comprehensive regulatory network for PM and promote its clinical translation in noninvasive diagnostic model development and targeted therapy.

## 5. Conclusion

This study identified KAZALD1, GLCE, and RPRD1B as potential diagnostic biomarkers for DN, with strong diagnostic performance. These biomarkers are involved in key biological pathways, including apoptosis, immune regulation, and metabolic processes. Immune cell analysis revealed their role in shaping the DN immune microenvironment. Regulatory network analysis highlighted their complex regulation by lncRNAs, miRNAs, and small molecules, suggesting potential therapeutic targets. Experimental validation confirmed the upregulation of KAZALD1 and RPRD1B in DN samples, while the expression pattern of GLCE requires further investigation. These findings provide valuable insights into DN pathogenesis and offer promising avenues for diagnosis and treatment.

## Ethics Statement

The data used in this study were ethically approved, and informed consent was obtained from the participants in the original research.

## Conflicts of Interest

The authors declare no conflicts of interest.

## Author Contributions

K.L. and S.Z. conceived and designed the study. S.Z. and H.A. participated in the data processing and bioinformatic analyses. R.W. and Z.W. were responsible for the validation of the analyses. S.Z. and H.W. wrote the original draft of the manuscript. S.Z. and M.L. performed the experimental validation. M.L. and T.L. provided technical guidance. S.Z. and H.A. are all first co‐authors.

## Funding

This study was funded by the Natural Science Foundation of Gansu Province, 10.13039/501100004775, 24JRRA1069.

## Supporting Information

Additional supporting information can be found online in the Supporting Information section.

## Supporting information


**Supporting Information 1** Figure S1: Protein interaction network diagram.

**Supporting Information 2** Table S1: The primer sequence of biomarkers.

**Supporting Information 3** Table S2: The GSEA pathway of biomarkers.

**Supporting Information 4** Table S3: The miRNA and lncRNA predicted by biomarkers.

## Data Availability

The study′s conclusions are backed by the data provided in the paper and its supporting information. Sequencing data are available via GEO, and for any additional relevant data not included in the paper or GEO, interested parties can request access from the corresponding authors, subject to reasonable conditions.
