# Supplementary material for: Identification and Verification of Biomarkers Related to Polyamine Metabolism in Diabetic Nephropathy
Source: J Diabetes Res. 2025 Dec 30;2025:9539734. doi: 10.1155/jdr/9539734 (PMC12767236; doi:10.1155/jdr/9539734)
Supplement: Supplementary file 1 — Supporting Information 1 Figure S1: Protein interaction network diagram. [file JDR-2025-9539734-s001.pdf]

S1

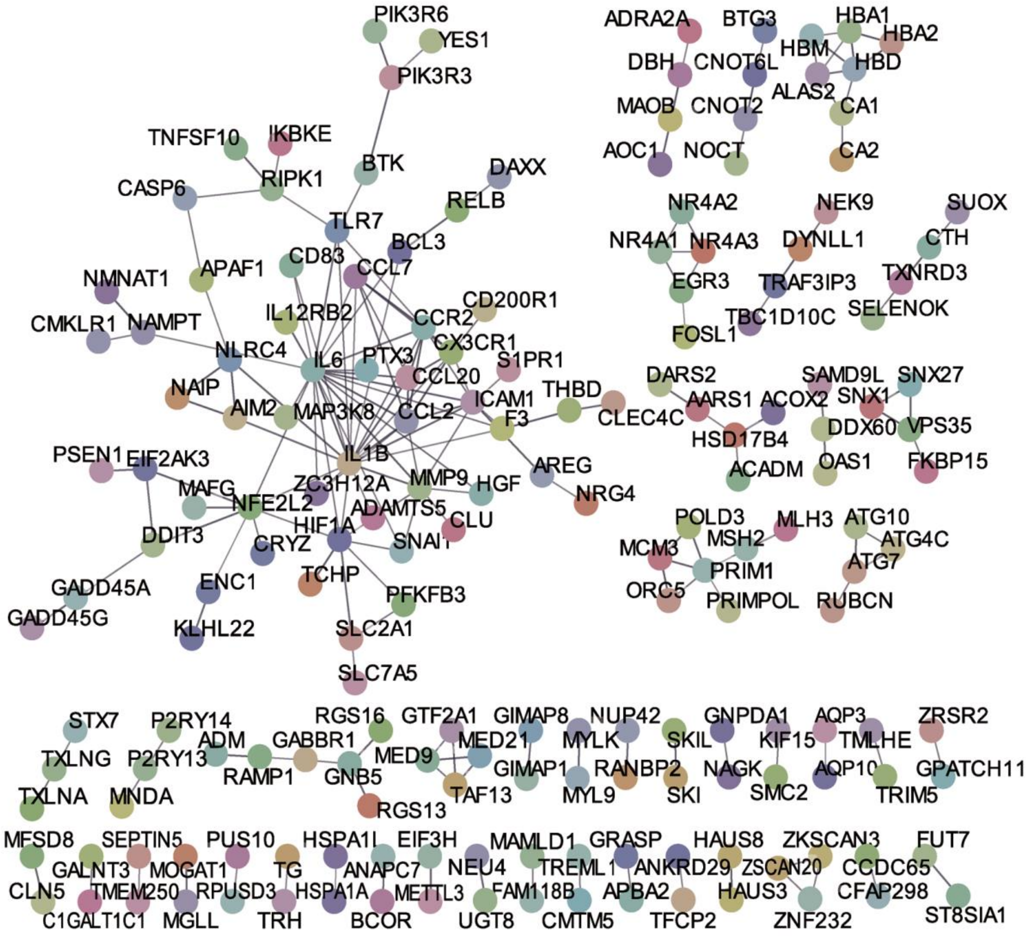

Figure S1 Protein interaction network diagram. Each dot represents a protein, and the connecting lines represent the interactions between two proteins, the darker the colour, the higher the connectivity
