# Supplementary material for: Identification and Verification of Biomarkers Related to Polyamine Metabolism in Diabetic Nephropathy
Source: J Diabetes Res. 2025 Dec 30;2025:9539734. doi: 10.1155/jdr/9539734 (PMC12767236; doi:10.1155/jdr/9539734)
Supplement: Supplementary file 2 — Supporting Information 2 Table S1: The primer sequence of biomarkers. [file JDR-2025-9539734-s004.docx]

| **Table S1 The primer sequence of biomarkers** | |
| --- | --- |
|  |  |
| **primer** | **sequence** |
| KAZALD1 F | CCTGCTAGCTTGACAGTGCT |
| KAZALD1 R | AACACTATAGCCGCTCACCC |
| GLCE F | CTGAGAAACCTCCTCACATAGAG |
| GLCE R | CAGCCACATTCGCCATAAAG |
| RPRD1B F | AGCAGCTGAAGCTGTCTATG |
| RPRD1B R | CATCCTCCTCCTCCTGAATTTG |
| GAPDH F | CGAAGGTGGAGTCAACGGATTT |
| GAPDH R | ATGGGTGGAATCATATTGGAAC |
