# Supplementary material for: Identification and Verification of Biomarkers Related to Polyamine Metabolism in Diabetic Nephropathy
Source: J Diabetes Res. 2025 Dec 30;2025:9539734. doi: 10.1155/jdr/9539734 (PMC12767236; doi:10.1155/jdr/9539734)
Supplement: Supplementary file 3 — Supporting Information 3 Table S2: The GSEA pathway of biomarkers. [file JDR-2025-9539734-s003.docx]

**Table S2 The GSEA pathway of biomarkers**

**KAZALD1**

| **ID** | **Description** | **setSize** | **enrichmentScore** | **NES** | **pvalue** | **p.adjust** | **qvalue** | **rank** | **leading_edge** | **core_enrichment** |  |
| --- | --- | --- | --- | --- | --- | --- | --- | --- | --- | --- | --- |
| KEGG_PRIMARY_IMMUNODEFICIENCY | KEGG_PRIMARY_IMMUNODEFICIENCY | KEGG_PRIMARY_IMMUNODEFICIENCY | 35 | -0.5333615 | -2.016472024 | 0.000101839 | 0.009971205 | 0.007757917 | 5206 | tags=74%, list=31%, signal=51% | 6891/5993/5788/926/57379/8517/959/916/973/8625/3561/64421/915/7535/23495/6890/3543/958/5994/920/930/3575/29760/4261/695/3932 |
| KEGG_CYTOKINE_CYTOKINE_RECEPTOR_INTERACTION | KEGG_CYTOKINE_CYTOKINE_RECEPTOR_INTERACTION | KEGG_CYTOKINE_CYTOKINE_RECEPTOR_INTERACTION | 251 | 0.322885969 | 1.633838709 | 0.000108383 | 0.009971205 | 0.007757917 | 3762 | tags=33%, list=23%, signal=26% | 8795/6364/2057/7422/58191/23529/3586/3625/84957/6347/3552/2921/3569/3553/3459/7040/51330/2920/53833/1233/7424/3556/8744/7852/2919/970/6354/1230/29949/7850/5159/7124/27242/4804/6368/356/115650/643/10344/5008/3442/3587/3559/8740/1950/3589/3605/6351/3554/3604/3443/3601/9547/3624/80301/944/3577/4352/7048/6362/3446/11009/64806/8771/116379/91/55801/50615/1440/7293/3458/3976/5154/1237/1437/8600/6352/2829/10663/58985/3558/8200/64109/1235 |
| KEGG_CIRCADIAN_RHYTHM_MAMMAL | KEGG_CIRCADIAN_RHYTHM_MAMMAL | KEGG_CIRCADIAN_RHYTHM_MAMMAL | 12 | 0.723362638 | 1.945540871 | 0.000378255 | 0.023199665 | 0.018050083 | 1425 | tags=50%, list=9%, signal=46% | 1453/8864/8553/1407/1454/1408 |
| KEGG_VALINE_LEUCINE_AND_ISOLEUCINE_DEGRADATION | KEGG_VALINE_LEUCINE_AND_ISOLEUCINE_DEGRADATION | KEGG_VALINE_LEUCINE_AND_ISOLEUCINE_DEGRADATION | 43 | -0.47572595 | -1.889429538 | 0.000570192 | 0.024934121 | 0.019399545 | 2056 | tags=37%, list=12%, signal=33% | 11112/38/4329/549/3155/223/224/1629/84693/5019/27034/594/3033/56922/26275/34 |
| KEGG_RENAL_CELL_CARCINOMA | KEGG_RENAL_CELL_CARCINOMA | KEGG_RENAL_CELL_CARCINOMA | 66 | 0.425551674 | 1.734027956 | 0.000677558 | 0.024934121 | 0.019399545 | 3362 | tags=36%, list=20%, signal=29% | 4893/3091/7422/7040/208/6513/7424/7428/5295/8453/2889/5290/2034/998/673/5291/56924/1398/5781/1387/2033/5605/2885/1399 |
| KEGG_P53_SIGNALING_PATHWAY | KEGG_P53_SIGNALING_PATHWAY | KEGG_P53_SIGNALING_PATHWAY | 65 | 0.434001654 | 1.763138971 | 0.000879234 | 0.026963168 | 0.020978208 | 3121 | tags=42%, list=19%, signal=34% | 8795/1647/842/8493/836/1026/6477/50484/1029/83667/898/4194/4616/9134/4193/54205/64065/10912/51512/1643/1111/143686/5366/7057/3486/85417/64393 |
| KEGG_PEROXISOME | KEGG_PEROXISOME | KEGG_PEROXISOME | 78 | -0.385551108 | -1.738848064 | 0.001164763 | 0.030616619 | 0.023820711 | 3365 | tags=37%, list=20%, signal=30% | 5195/10901/4358/8504/8443/54677/189/55825/10654/51/23600/5189/3155/26061/7498/225/5830/8310/5826/847/10455/83594/23417/2053/3417/196743/3295/10478/8799 |
| KEGG_MISMATCH_REPAIR | KEGG_MISMATCH_REPAIR | KEGG_MISMATCH_REPAIR | 23 | -0.561141408 | -1.920113349 | 0.001746794 | 0.035712236 | 0.027785264 | 2871 | tags=48%, list=17%, signal=40% | 4437/4292/3978/5985/29935/57804/6117/6118/5984/10714/4436 |
| KEGG_PATHWAYS_IN_CANCER | KEGG_PATHWAYS_IN_CANCER | KEGG_PATHWAYS_IN_CANCER | 321 | 0.278724431 | 1.445636696 | 0.001609968 | 0.035712236 | 0.027785264 | 3518 | tags=28%, list=21%, signal=22% | 4088/4893/3091/3914/7422/842/4318/836/1026/8030/5337/4824/9618/861/5914/3569/7471/10342/7040/208/6513/1029/7424/7428/5743/5295/2254/4149/83593/1869/8453/7855/898/5970/5290/8324/5159/329/2034/7187/998/9134/7704/4193/54205/4312/7481/673/652/5291/2248/356/3674/1398/7472/1950/4089/3480/9063/7849/1284/8817/8325/5727/7048/1387/26281/7476/2737/2033/1499/5605/598/4791/8322/2885/3913/7189/2335/5599/3675/1399/8326/5154/26291/5468/25/637/3909 |
| KEGG_GLYCOSAMINOGLYCAN_BIOSYNTHESIS_KERATAN_SULFATE | KEGG_GLYCOSAMINOGLYCAN_BIOSYNTHESIS_KERATAN_SULFATE | KEGG_GLYCOSAMINOGLYCAN_BIOSYNTHESIS_KERATAN_SULFATE | 14 | 0.661729791 | 1.879228029 | 0.002335098 | 0.039059815 | 0.030389787 | 4346 | tags=71%, list=26%, signal=53% | 2683/93010/8702/10678/8704/10164/2530/6482/8534/6487 |
| KEGG_ENDOCYTOSIS | KEGG_ENDOCYTOSIS | KEGG_ENDOCYTOSIS | 174 | 0.315240951 | 1.51586968 | 0.002320907 | 0.039059815 | 0.030389787 | 2251 | tags=24%, list=14%, signal=21% | 30844/79720/51100/7037/83737/5337/128866/55048/5878/57154/80223/6455/7852/84552/25978/5868/2149/10938/8027/84440/1212/998/9146/4193/1759/9922/7251/55738/116984/6714/3559/1950/200576/3480/26056/84313/1175/3306/23327/29924/2870/9101 |
| KEGG_NEUROTROPHIN_SIGNALING_PATHWAY | KEGG_NEUROTROPHIN_SIGNALING_PATHWAY | KEGG_NEUROTROPHIN_SIGNALING_PATHWAY | 125 | 0.333035291 | 1.526849683 | 0.002596739 | 0.039816663 | 0.03097864 | 2117 | tags=26%, list=13%, signal=22% | 5598/4893/8767/396/5663/7534/208/10019/9261/4794/468/5295/5600/817/2889/11108/5970/5290/998/10971/4804/8660/673/5291/2309/356/7532/4793/1398/10603/5781/3656 |
| KEGG_TGF_BETA_SIGNALING_PATHWAY | KEGG_TGF_BETA_SIGNALING_PATHWAY | KEGG_TGF_BETA_SIGNALING_PATHWAY | 85 | 0.362752573 | 1.548880232 | 0.003051052 | 0.043184127 | 0.033598634 | 3825 | tags=35%, list=23%, signal=27% | 4088/4092/3625/5515/57154/7040/3397/3399/4091/6198/7124/4052/5519/652/4086/9372/4089/8646/7057/3624/7048/1387/3398/2033/3458/6667/654/8200/7027/7060 |
| KEGG_HOMOLOGOUS_RECOMBINATION | KEGG_HOMOLOGOUS_RECOMBINATION | KEGG_HOMOLOGOUS_RECOMBINATION | 26 | -0.522938748 | -1.848751189 | 0.003391006 | 0.044028761 | 0.034255786 | 2774 | tags=42%, list=17%, signal=35% | 29935/57804/7516/5892/10111/6117/8940/6118/10714/80198/5893 |
| KEGG_FATTY_ACID_METABOLISM | KEGG_FATTY_ACID_METABOLISM | KEGG_FATTY_ACID_METABOLISM | 41 | -0.429138048 | -1.687344207 | 0.003589301 | 0.044028761 | 0.034255786 | 3362 | tags=34%, list=20%, signal=27% | 127/1892/501/1375/38/51/8310/223/224/2639/10455/3033/128/34 |
| KEGG_BLADDER_CANCER | KEGG_BLADDER_CANCER | KEGG_BLADDER_CANCER | 40 | 0.458870143 | 1.693931983 | 0.003948393 | 0.044782691 | 0.034842368 | 2442 | tags=30%, list=15%, signal=26% | 4893/7422/4318/1026/1029/7424/1869/4193/4312/673/1950/7057 |
| KEGG_JAK_STAT_SIGNALING_PATHWAY | KEGG_JAK_STAT_SIGNALING_PATHWAY | KEGG_JAK_STAT_SIGNALING_PATHWAY | 146 | 0.318511324 | 1.494415052 | 0.004137531 | 0.044782691 | 0.034842368 | 3756 | tags=32%, list=23%, signal=25% | 2057/23529/3586/200734/30837/10253/3569/3459/10252/53833/208/5295/122809/6775/5290/8027/29949/5291/8651/81848/5008/3442/3587/3559/5781/3589/9063/3443/3601/4352/1387/3446/11009/2033/116379/598/55801/50615/1440/2885/3458/9021/3976/1437/58985/3558/64109 |
| KEGG_COMPLEMENT_AND_COAGULATION_CASCADES | KEGG_COMPLEMENT_AND_COAGULATION_CASCADES | KEGG_COMPLEMENT_AND_COAGULATION_CASCADES | 68 | 0.394034955 | 1.613596776 | 0.004404675 | 0.045025569 | 0.035031335 | 5192 | tags=49%, list=31%, signal=33% | 7056/728/5329/5328/2152/715/7035/2160/3053/2149/2161/5104/5265/718/7450/2159/629/716/5627/735/730/3426/624/2158/713/1604/2243/719/2/721/5327/731/2155 |

**GLCE**

| **ID** | **Description** | **setSize** | **enrichmentScore** | **NES** | **pvalue** | **p.adjust** | **qvalue** | **rank** | **leading_edge** | **core_enrichment** |  |
| --- | --- | --- | --- | --- | --- | --- | --- | --- | --- | --- | --- |
| KEGG_PEROXISOME | KEGG_PEROXISOME | KEGG_PEROXISOME | 78 | 0.508753683 | 2.003944238 | 6.66E-06 | 0.000613007 | 0.00048746 | 4326 | tags=53%, list=26%, signal=39% | 3295/8799/3417/196743/10478/10455/3155/23417/51/8310/847/10654/5052/8443/8504/55825/23600/4358/5826/26061/225/5189/2053/10901/5195/5830/83594/215/55711/8800/11264/51703/1891/2182/10005/30/5824/5190/55670/9409/4598 |
| KEGG_CYTOKINE_CYTOKINE_RECEPTOR_INTERACTION | KEGG_CYTOKINE_CYTOKINE_RECEPTOR_INTERACTION | KEGG_CYTOKINE_CYTOKINE_RECEPTOR_INTERACTION | 251 | -0.330859776 | -1.710155097 | 3.40E-06 | 0.000613007 | 0.00048746 | 3416 | tags=33%, list=21%, signal=27% | 6376/4233/55801/6348/5159/8771/8792/944/4283/2829/6352/116379/7293/2323/3563/83729/3589/2324/7850/3581/64109/414062/3587/3442/3554/3976/56477/10803/3604/9547/658/5154/115650/1233/10344/1235/53833/80301/1230/1950/2833/8200/27242/643/3601/1270/7424/1896/3624/27190/29949/6351/3459/7048/8740/3569/51330/970/4804/6368/50615/7422/3586/7040/2057/5008/23529/3625/7124/3556/6354/6347/2921/84957/2919/2920/7852/58191/3552/8744/6364/8795/3553 |
| KEGG_VALINE_LEUCINE_AND_ISOLEUCINE_DEGRADATION | KEGG_VALINE_LEUCINE_AND_ISOLEUCINE_DEGRADATION | KEGG_VALINE_LEUCINE_AND_ISOLEUCINE_DEGRADATION | 43 | 0.54317277 | 1.909118206 | 0.000136372 | 0.008364132 | 0.006651111 | 4071 | tags=63%, list=25%, signal=47% | 34/26275/3033/3155/56922/38/549/84693/27034/5019/11112/1892/224/223/4329/1629/587/594/5095/593/3032/219/39/30/64087/18/5096 |
| KEGG_PATHWAYS_IN_CANCER | KEGG_PATHWAYS_IN_CANCER | KEGG_PATHWAYS_IN_CANCER | 321 | -0.277205846 | -1.474058927 | 0.000183203 | 0.008427348 | 0.006701381 | 2564 | tags=24%, list=16%, signal=21% | 2885/4089/1398/3909/1499/6469/10297/8323/1387/2122/5154/3674/329/1399/3480/2033/2260/1029/8326/652/7187/330/27006/2308/89780/1950/8325/5467/7481/7424/8324/7189/25/7476/7704/10342/7428/4193/7048/2248/3569/83593/998/9063/1869/4791/208/7422/6513/4149/2034/5290/861/7471/8453/8817/5970/5337/842/8030/7040/2254/5914/5599/5743/4824/836/1026/4893/898/4318/3091/5295/9618/54205/4088/3914 |
| KEGG_CITRATE_CYCLE_TCA_CYCLE | KEGG_CITRATE_CYCLE_TCA_CYCLE | KEGG_CITRATE_CYCLE_TCA_CYCLE | 30 | 0.590179079 | 1.928170788 | 0.000378291 | 0.013921113 | 0.011069993 | 2565 | tags=47%, list=16%, signal=39% | 3417/48/4191/5106/8802/5160/5162/6391/2271/50/4190/6392/3419/47 |
| KEGG_CIRCADIAN_RHYTHM_MAMMAL | KEGG_CIRCADIAN_RHYTHM_MAMMAL | KEGG_CIRCADIAN_RHYTHM_MAMMAL | 12 | -0.719125609 | -1.961503283 | 0.000984866 | 0.021784188 | 0.017322667 | 2212 | tags=67%, list=13%, signal=58% | 9572/5187/1454/8553/1408/1407/8864/1453 |
| KEGG_MISMATCH_REPAIR | KEGG_MISMATCH_REPAIR | KEGG_MISMATCH_REPAIR | 23 | 0.611940841 | 1.850210127 | 0.000717663 | 0.021784188 | 0.017322667 | 3827 | tags=52%, list=23%, signal=40% | 4436/10714/6118/6117/5984/57804/5985/5424/3978/4292/5982/4437 |
| KEGG_PENTOSE_AND_GLUCURONATE_INTERCONVERSIONS | KEGG_PENTOSE_AND_GLUCURONATE_INTERCONVERSIONS | KEGG_PENTOSE_AND_GLUCURONATE_INTERCONVERSIONS | 20 | 0.613577905 | 1.801648664 | 0.001775885 | 0.021784188 | 0.017322667 | 1969 | tags=35%, list=12%, signal=31% | 7358/7360/51084/6120/7367/231/7366 |
| KEGG_STARCH_AND_SUCROSE_METABOLISM | KEGG_STARCH_AND_SUCROSE_METABOLISM | KEGG_STARCH_AND_SUCROSE_METABOLISM | 39 | 0.523349819 | 1.787124137 | 0.001579021 | 0.021784188 | 0.017322667 | 5202 | tags=51%, list=31%, signal=35% | 2632/7358/178/7360/55276/2997/7367/7366/5836/57733/3098/5167/283209/5236/54490/2548/5169/278/5837/54576 |
| KEGG_DNA_REPLICATION | KEGG_DNA_REPLICATION | KEGG_DNA_REPLICATION | 36 | 0.525697225 | 1.772732839 | 0.001235563 | 0.021784188 | 0.017322667 | 2776 | tags=44%, list=17%, signal=37% | 4172/5557/10714/79621/6118/6117/5984/57804/5422/5985/84153/23649/10535/5424/3978/1763 |
| KEGG_NUCLEOTIDE_EXCISION_REPAIR | KEGG_NUCLEOTIDE_EXCISION_REPAIR | KEGG_NUCLEOTIDE_EXCISION_REPAIR | 43 | 0.503361379 | 1.769190996 | 0.001262365 | 0.021784188 | 0.017322667 | 3994 | tags=56%, list=24%, signal=42% | 2965/8451/10714/6118/6117/5984/57804/9978/5985/1642/2068/7507/2968/5424/3978/8450/2967/4331/2072/5982/2074/7508/404672/5426 |
| KEGG_P53_SIGNALING_PATHWAY | KEGG_P53_SIGNALING_PATHWAY | KEGG_P53_SIGNALING_PATHWAY | 65 | -0.414070411 | -1.742858387 | 0.001147325 | 0.021784188 | 0.017322667 | 1838 | tags=31%, list=11%, signal=27% | 64065/83667/25898/1111/4193/50484/10912/5366/4194/143686/842/4616/836/1026/6477/898/1647/54205/8493/8795 |
| KEGG_ANTIGEN_PROCESSING_AND_PRESENTATION | KEGG_ANTIGEN_PROCESSING_AND_PRESENTATION | KEGG_ANTIGEN_PROCESSING_AND_PRESENTATION | 77 | 0.420023084 | 1.651362427 | 0.001659463 | 0.021784188 | 0.017322667 | 4692 | tags=45%, list=28%, signal=33% | 3303/4801/4802/3308/6890/4261/3305/3111/8625/3312/1385/920/1520/10197/5993/3310/3821/5720/3108/5721/3123/3109/821/3127/5994/3822/3823/3115/3134/3112/3122/567/1508/3106/3125 |
| KEGG_PYRIMIDINE_METABOLISM | KEGG_PYRIMIDINE_METABOLISM | KEGG_PYRIMIDINE_METABOLISM | 92 | 0.409736083 | 1.648737198 | 0.001331703 | 0.021784188 | 0.017322667 | 3324 | tags=37%, list=20%, signal=30% | 1841/5557/87178/10714/5437/84265/1723/1633/10201/5435/1806/5436/55703/57804/51728/29922/4907/5422/1854/790/3704/23649/30833/953/10623/84618/5424/318/6241/4832/84172/11128/56474/171568 |
| KEGG_PURINE_METABOLISM | KEGG_PURINE_METABOLISM | KEGG_PURINE_METABOLISM | 146 | 0.35505047 | 1.537832559 | 0.001447453 | 0.021784188 | 0.017322667 | 3324 | tags=31%, list=20%, signal=25% | 113/5557/87178/11164/10714/5147/5437/5150/53343/84265/1633/204/471/272/10201/5435/9061/2618/5436/55703/57804/51728/29922/4907/5198/5422/51292/5634/3704/23649/30833/953/10623/84618/5424/318/3615/5471/6241/4832/158/26289/84172/11128/171568 |
| KEGG_PRIMARY_IMMUNODEFICIENCY | KEGG_PRIMARY_IMMUNODEFICIENCY | KEGG_PRIMARY_IMMUNODEFICIENCY | 35 | 0.52231052 | 1.752880952 | 0.002006717 | 0.023077246 | 0.018350899 | 4570 | tags=66%, list=28%, signal=48% | 6890/29760/958/4261/8517/3932/695/8625/920/5993/64421/3543/5788/3575/57379/3561/5896/5994/959/7535/915/930/100 |
| KEGG_PROPANOATE_METABOLISM | KEGG_PROPANOATE_METABOLISM | KEGG_PROPANOATE_METABOLISM | 31 | 0.543286243 | 1.782854239 | 0.002235803 | 0.023429406 | 0.018630935 | 4071 | tags=52%, list=25%, signal=39% | 34/26275/23417/38/84693/8802/1892/224/223/4329/5095/219/39/3945/18/5096 |
| KEGG_ERBB_SIGNALING_PATHWAY | KEGG_ERBB_SIGNALING_PATHWAY | KEGG_ERBB_SIGNALING_PATHWAY | 86 | -0.368345112 | -1.62527955 | 0.002292007 | 0.023429406 | 0.018630935 | 2747 | tags=28%, list=17%, signal=23% | 5582/2885/1398/685/1399/10718/1978/1950/25759/145957/25/6714/817/27/208/5290/6198/2069/5599/1026/4893/374/5295/1839 |
| KEGG_LYSOSOME | KEGG_LYSOSOME | KEGG_LYSOSOME | 118 | 0.364860045 | 1.530595578 | 0.002660448 | 0.025764341 | 0.020487663 | 4390 | tags=38%, list=27%, signal=28% | 1203/4074/2720/256471/8905/285362/1213/1497/8763/8218/23431/4669/3920/51606/9179/1520/2517/54/4126/84572/8943/3074/51172/1211/3988/175/8907/4668/6609/23163/26985/1075/23659/5538/535/3073/22901/1777/1519/5476/1512/10577/2588/1508/3916 |
| KEGG_FATTY_ACID_METABOLISM | KEGG_FATTY_ACID_METABOLISM | KEGG_FATTY_ACID_METABOLISM | 41 | 0.483137176 | 1.683827975 | 0.002816348 | 0.025910399 | 0.020603807 | 3814 | tags=44%, list=23%, signal=34% | 34/3033/10455/51/38/128/8310/1892/224/223/1632/2639/3032/219/51703/39/2182/30 |
| KEGG_NEUROTROPHIN_SIGNALING_PATHWAY | KEGG_NEUROTROPHIN_SIGNALING_PATHWAY | KEGG_NEUROTROPHIN_SIGNALING_PATHWAY | 125 | -0.313369704 | -1.490833046 | 0.003020229 | 0.026324975 | 0.020933475 | 2564 | tags=26%, list=16%, signal=22% | 2885/2889/1398/11213/1399/11108/7532/2309/25759/4794/5781/7189/25/5600/7534/817/4793/4804/998/8660/208/5290/5970/468/10019/9261/5599/4893/5663/396/8767/5295/5598 |
| KEGG_MAPK_SIGNALING_PATHWAY | KEGG_MAPK_SIGNALING_PATHWAY | KEGG_MAPK_SIGNALING_PATHWAY | 261 | -0.268255739 | -1.388535299 | 0.003147551 | 0.026324975 | 0.020933475 | 2262 | tags=21%, list=14%, signal=18% | 2122/5154/1399/5319/2260/23162/27006/1950/3306/1852/4137/8491/7189/2318/11072/22800/5600/1847/7048/5534/784/2248/3727/80824/998/2005/4791/208/4149/10912/3164/1326/8817/5970/468/5778/1844/2768/7040/2254/9261/5599/4616/836/5971/7124/11221/4893/1647/3552/5598/1846/1649/1850/3553 |
| KEGG_GLYCOSAMINOGLYCAN_BIOSYNTHESIS_KERATAN_SULFATE | KEGG_GLYCOSAMINOGLYCAN_BIOSYNTHESIS_KERATAN_SULFATE | KEGG_GLYCOSAMINOGLYCAN_BIOSYNTHESIS_KERATAN_SULFATE | 14 | -0.639664062 | -1.850857833 | 0.004386172 | 0.035089378 | 0.02790288 | 2572 | tags=57%, list=16%, signal=48% | 10164/2530/8704/10678/6487/8702/2683/93010 |
| KEGG_ONE_CARBON_POOL_BY_FOLATE | KEGG_ONE_CARBON_POOL_BY_FOLATE | KEGG_ONE_CARBON_POOL_BY_FOLATE | 17 | 0.624747552 | 1.766330106 | 0.005619758 | 0.043084812 | 0.034260806 | 2971 | tags=65%, list=18%, signal=53% | 471/123263/2618/6472/4548/275/10588/4522/1719/6470/4524 |
| KEGG_BASE_EXCISION_REPAIR | KEGG_BASE_EXCISION_REPAIR | KEGG_BASE_EXCISION_REPAIR | 33 | 0.498815247 | 1.653667943 | 0.006674046 | 0.04912098 | 0.039060734 | 4050 | tags=55%, list=24%, signal=41% | 142/143/10714/23583/4913/4968/7515/27301/57804/8930/5424/3978/4350/3146/4595/27343/5426/328 |

**RPRD1B**

| **ID** | **Description** | **setSize** | **enrichmentScore** | **NES** | **pvalue** | **p.adjust** | **qvalue** | **rank** | **leading_edge** | **core_enrichment** |  |
| --- | --- | --- | --- | --- | --- | --- | --- | --- | --- | --- | --- |
| KEGG_CYTOKINE_CYTOKINE_RECEPTOR_INTERACTION | KEGG_CYTOKINE_CYTOKINE_RECEPTOR_INTERACTION | KEGG_CYTOKINE_CYTOKINE_RECEPTOR_INTERACTION | 251 | 0.318399432 | 1.678511899 | 4.63E-06 | 0.000852359 | 0.000668038 | 2814 | tags=29%, list=17%, signal=24% | 8795/3552/3553/2057/84957/6354/6364/58191/7852/8744/6347/970/2919/2921/51330/3569/2920/3625/643/7124/3586/3556/23529/7422/5008/29949/6351/7048/7040/3624/5154/8771/50615/1230/53833/4804/3459/7424/1233/1950/6368/2833/115650/8740/3589/3976/5159/1235/3601/7850/27190/80301/9547/10344/3604/55801/3563/1270/7046/3558/658/8200/6376/91/414062/3442/1896/10803/3605/27242/9966/6367 |
| KEGG_PEROXISOME | KEGG_PEROXISOME | KEGG_PEROXISOME | 78 | -0.487139517 | -1.971150829 | 1.15E-05 | 0.001061127 | 0.000831661 | 4501 | tags=55%, list=27%, signal=40% | 11264/1384/10005/3418/7498/5189/54677/4598/9409/51703/373156/26061/5195/5826/5824/30/215/83594/8800/8504/1891/55711/5830/10901/5052/225/2053/23600/55825/4358/8443/3155/8310/10455/847/51/10654/196743/10478/23417/3417/8799/3295 |
| KEGG_VALINE_LEUCINE_AND_ISOLEUCINE_DEGRADATION | KEGG_VALINE_LEUCINE_AND_ISOLEUCINE_DEGRADATION | KEGG_VALINE_LEUCINE_AND_ISOLEUCINE_DEGRADATION | 43 | -0.567997247 | -2.042500215 | 1.94E-05 | 0.001191082 | 0.000933514 | 4209 | tags=70%, list=25%, signal=52% | 3030/18/10449/217/3712/39/593/5096/219/3032/30/5095/594/587/1629/84693/11112/38/223/5019/549/56922/224/3155/1892/4329/3033/27034/26275/34 |
| KEGG_PROPANOATE_METABOLISM | KEGG_PROPANOATE_METABOLISM | KEGG_PROPANOATE_METABOLISM | 31 | -0.580481507 | -1.962042702 | 0.000328238 | 0.014798777 | 0.011598584 | 4209 | tags=58%, list=25%, signal=43% | 3030/18/3945/217/39/5096/219/5095/84693/38/223/224/1892/4329/8802/23417/26275/34 |
| KEGG_MISMATCH_REPAIR | KEGG_MISMATCH_REPAIR | KEGG_MISMATCH_REPAIR | 23 | -0.626119194 | -1.9535352 | 0.000420141 | 0.014798777 | 0.011598584 | 4780 | tags=74%, list=29%, signal=53% | 5425/5983/29935/27030/4437/5395/5985/5424/4292/5982/57804/6117/5984/3978/6118/10714/4436 |
| KEGG_P53_SIGNALING_PATHWAY | KEGG_P53_SIGNALING_PATHWAY | KEGG_P53_SIGNALING_PATHWAY | 65 | 0.424926184 | 1.776085487 | 0.000482569 | 0.014798777 | 0.011598584 | 2341 | tags=37%, list=14%, signal=32% | 6477/8795/836/8493/898/54205/1647/842/50484/4616/143686/1026/10912/4194/5366/4193/1111/1029/64065/3486/2810/56475/83667/1643 |
| KEGG_ANTIGEN_PROCESSING_AND_PRESENTATION | KEGG_ANTIGEN_PROCESSING_AND_PRESENTATION | KEGG_ANTIGEN_PROCESSING_AND_PRESENTATION | 77 | -0.436178983 | -1.768527713 | 0.000614746 | 0.016159026 | 0.012664683 | 5455 | tags=57%, list=33%, signal=38% | 4049/8302/3452/10437/4800/972/3122/3135/5993/3107/3125/3823/3126/3105/5721/1508/3109/3108/3133/3115/3134/3127/3106/3123/3821/3822/5994/821/1385/1520/8625/5720/3310/4261/3312/3111/920/6890/10197/3305/4802/3308/4801/3303 |
| KEGG_CITRATE_CYCLE_TCA_CYCLE | KEGG_CITRATE_CYCLE_TCA_CYCLE | KEGG_CITRATE_CYCLE_TCA_CYCLE | 30 | -0.572480857 | -1.916123862 | 0.000881546 | 0.016722181 | 0.013106058 | 3473 | tags=53%, list=21%, signal=42% | 6392/4967/5091/4190/5160/47/3419/2271/6391/50/5162/5106/8802/4191/48/3417 |
| KEGG_PRIMARY_IMMUNODEFICIENCY | KEGG_PRIMARY_IMMUNODEFICIENCY | KEGG_PRIMARY_IMMUNODEFICIENCY | 35 | -0.538592941 | -1.858937221 | 0.000910685 | 0.016722181 | 0.013106058 | 4386 | tags=69%, list=27%, signal=50% | 5993/5896/930/916/959/915/5788/100/57379/64421/7535/5994/3543/3561/29760/8625/4261/920/8517/3575/3932/6890/958/695 |
| KEGG_FATTY_ACID_METABOLISM | KEGG_FATTY_ACID_METABOLISM | KEGG_FATTY_ACID_METABOLISM | 41 | -0.510743502 | -1.827475716 | 0.000794267 | 0.016722181 | 0.013106058 | 3775 | tags=46%, list=23%, signal=36% | 51703/10449/217/39/219/3032/1632/30/38/223/224/1892/2639/8310/10455/3033/51/128/34 |
| KEGG_COMPLEMENT_AND_COAGULATION_CASCADES | KEGG_COMPLEMENT_AND_COAGULATION_CASCADES | KEGG_COMPLEMENT_AND_COAGULATION_CASCADES | 68 | 0.402580053 | 1.699986807 | 0.000999696 | 0.016722181 | 0.013106058 | 4727 | tags=46%, list=29%, signal=33% | 7056/2152/5329/7035/715/5328/728/716/2161/3053/731/5327/2160/624/2159/5104/2243/2155/7450/2149/2165/2162/730/721/719/5627/1604/718/3818/733/5624 |
| KEGG_RENAL_CELL_CARCINOMA | KEGG_RENAL_CELL_CARCINOMA | KEGG_RENAL_CELL_CARCINOMA | 66 | 0.39432612 | 1.658678155 | 0.001146188 | 0.017574877 | 0.01377436 | 1979 | tags=29%, list=12%, signal=25% | 3091/4893/5295/8453/6513/5290/208/5781/7422/7428/7040/2034/1387/7424/998/673/1399/2889/8503 |
| KEGG_BUTANOATE_METABOLISM | KEGG_BUTANOATE_METABOLISM | KEGG_BUTANOATE_METABOLISM | 33 | -0.528735817 | -1.806654926 | 0.001464756 | 0.019251081 | 0.01508809 | 4209 | tags=55%, list=25%, signal=41% | 3030/18/6296/217/39/219/5160/7915/65985/5162/38/223/5019/224/3155/1892/622/3033 |
| KEGG_PATHWAYS_IN_CANCER | KEGG_PATHWAYS_IN_CANCER | KEGG_PATHWAYS_IN_CANCER | 321 | 0.25644109 | 1.378693137 | 0.001457695 | 0.019251081 | 0.01508809 | 1992 | tags=20%, list=12%, signal=18% | 836/8030/3914/2254/3091/9063/898/4824/54205/4318/9618/4893/861/5295/4088/842/8453/6513/5290/208/5743/1026/3569/5599/1869/5914/4149/7422/10342/7428/7048/5337/4193/7040/7471/2034/25/5154/2248/1029/8817/330/4089/1387/8326/5970/7424/998/83593/329/5467/673/7704/1950/1399/8324/7476/10297/3909/5159/4791/8503/5582 |
| KEGG_ERBB_SIGNALING_PATHWAY | KEGG_ERBB_SIGNALING_PATHWAY | KEGG_ERBB_SIGNALING_PATHWAY | 86 | 0.368733154 | 1.639235007 | 0.001773291 | 0.020392852 | 0.015982956 | 2388 | tags=28%, list=14%, signal=24% | 6198/817/4893/5295/374/5290/208/1026/5599/1839/27/2069/145957/25/6714/673/1950/1399/1978/2065/8503/5582/10718/685 |
| KEGG_LYSOSOME | KEGG_LYSOSOME | KEGG_LYSOSOME | 118 | -0.365084797 | -1.571235914 | 0.001702007 | 0.020392852 | 0.015982956 | 5549 | tags=51%, list=34%, signal=34% | 1201/1513/4125/950/2760/55353/8722/535/3916/10577/6272/53/2548/537/23163/5660/9476/1075/27074/22901/1512/3988/5476/1200/4669/1519/1508/54/5538/175/1211/3073/26985/1777/23659/3920/51606/2588/8943/3074/1520/4668/2517/8907/23431/51172/4126/9179/6609/84572/1497/8763/1213/8218/8905/2720/256471/285362/4074/1203 |
| KEGG_AMINOACYL_TRNA_BIOSYNTHESIS | KEGG_AMINOACYL_TRNA_BIOSYNTHESIS | KEGG_AMINOACYL_TRNA_BIOSYNTHESIS | 22 | -0.586406778 | -1.822471637 | 0.001932088 | 0.020537638 | 0.016096432 | 2778 | tags=55%, list=17%, signal=45% | 10667/57038/25973/57176/57505/80222/123263/124454/2193/79587/55699/55157 |
| KEGG_STARCH_AND_SUCROSE_METABOLISM | KEGG_STARCH_AND_SUCROSE_METABOLISM | KEGG_STARCH_AND_SUCROSE_METABOLISM | 39 | -0.512815484 | -1.811669682 | 0.00217966 | 0.020537638 | 0.016096432 | 5334 | tags=51%, list=32%, signal=35% | 278/54576/7363/2548/5837/7367/5169/3101/54490/5236/11181/7366/5836/3098/55276/2997/178/2632/7360/7358 |
| KEGG_PYRIMIDINE_METABOLISM | KEGG_PYRIMIDINE_METABOLISM | KEGG_PYRIMIDINE_METABOLISM | 92 | -0.390818428 | -1.620068333 | 0.002288929 | 0.020537638 | 0.016096432 | 3230 | tags=36%, list=20%, signal=29% | 4832/5424/953/5422/11128/84618/6241/1635/3704/56474/318/57804/5436/23649/1854/171568/790/4907/51728/29922/5435/55703/1633/10623/1723/84265/5437/1806/10201/1841/10714/87178/5557 |
| KEGG_PURINE_METABOLISM | KEGG_PURINE_METABOLISM | KEGG_PURINE_METABOLISM | 146 | -0.342467005 | -1.521064117 | 0.00234397 | 0.020537638 | 0.016096432 | 3230 | tags=30%, list=20%, signal=24% | 4832/3615/5424/953/5422/11128/84618/6241/3704/318/57804/5471/5158/26289/158/5436/9061/23649/5634/5198/171568/4907/51728/29922/5435/55703/272/1633/51292/53343/10623/5150/204/84265/5437/471/10201/2618/5147/10714/87178/11164/5557/113 |
| KEGG_MAPK_SIGNALING_PATHWAY | KEGG_MAPK_SIGNALING_PATHWAY | KEGG_MAPK_SIGNALING_PATHWAY | 261 | 0.269601552 | 1.430829756 | 0.002067313 | 0.020537638 | 0.016096432 | 2129 | tags=22%, list=13%, signal=20% | 1649/1850/3552/1846/836/5971/2254/3553/1647/4893/11221/5598/3727/4616/208/5599/10912/5600/7124/4149/1326/3164/784/8491/23162/7048/5778/7040/9261/2768/3306/468/5534/5154/2248/8817/22800/1852/4137/5970/998/1844/80824/2005/2318/673/1950/1399/5319/11072/5159/4791/7850/5582/1847/55970/5320/5495 |
| KEGG_CIRCADIAN_RHYTHM_MAMMAL | KEGG_CIRCADIAN_RHYTHM_MAMMAL | KEGG_CIRCADIAN_RHYTHM_MAMMAL | 12 | 0.675728606 | 1.839264454 | 0.003721923 | 0.031128812 | 0.024397295 | 1364 | tags=50%, list=8%, signal=46% | 1453/8864/1407/1408/1454/8553 |
| KEGG_SULFUR_METABOLISM | KEGG_SULFUR_METABOLISM | KEGG_SULFUR_METABOLISM | 12 | -0.693854816 | -1.838454771 | 0.004216227 | 0.032324407 | 0.025334346 | 2213 | tags=50%, list=13%, signal=43% | 6817/9061/6821/6799/445329/10380 |
| KEGG_PENTOSE_AND_GLUCURONATE_INTERCONVERSIONS | KEGG_PENTOSE_AND_GLUCURONATE_INTERCONVERSIONS | KEGG_PENTOSE_AND_GLUCURONATE_INTERCONVERSIONS | 20 | -0.5780846 | -1.759138027 | 0.004104093 | 0.032324407 | 0.025334346 | 5123 | tags=55%, list=31%, signal=38% | 54576/7363/27294/7367/54490/7366/6120/231/51084/7360/7358 |
| KEGG_DNA_REPLICATION | KEGG_DNA_REPLICATION | KEGG_DNA_REPLICATION | 36 | -0.479525729 | -1.671346606 | 0.005399687 | 0.039741694 | 0.031147667 | 4780 | tags=58%, list=29%, signal=42% | 5983/29935/4174/1763/5427/84153/5985/5424/5422/5982/57804/6117/5984/3978/23649/10535/79621/6118/10714/4172/5557 |
| KEGG_PYRUVATE_METABOLISM | KEGG_PYRUVATE_METABOLISM | KEGG_PYRUVATE_METABOLISM | 39 | -0.483837722 | -1.709297316 | 0.006806711 | 0.048170573 | 0.037753824 | 3789 | tags=46%, list=23%, signal=36% | 3945/217/39/219/5091/4190/9380/5160/3029/97/231/5162/38/223/224/5106/4200/4191 |
