# Supplementary material for: Identification and Verification of Biomarkers Related to Polyamine Metabolism in Diabetic Nephropathy
Source: J Diabetes Res. 2025 Dec 30;2025:9539734. doi: 10.1155/jdr/9539734 (PMC12767236; doi:10.1155/jdr/9539734)
Supplement: Supplementary file 4 — Supporting Information 4 Table S3: The miRNA and lncRNA predicted by biomarkers. [file JDR-2025-9539734-s002.docx]

| **Table S3 The miRNA and lncRNA predicted by biomarkers** | | | | | |
| --- | --- | --- | --- | --- | --- |
|  | **miRNA** | **miID** | **lncRNA** | **pancancerNum** | **Gene_id** |
| 1 | hsa-miR-892c-5p | MIMAT0025857 | AF127577.4 | 6 | KAZALD1 |
| 2 | hsa-miR-892c-5p | MIMAT0025857 | AC093297.2 | 5 | KAZALD1 |
| 3 | hsa-miR-892c-5p | MIMAT0025857 | PPP3CB-AS1 | 5 | KAZALD1 |
| 4 | hsa-miR-892c-5p | MIMAT0025857 | PPP3CB-AS1 | 5 | KAZALD1 |
| 5 | hsa-miR-892c-5p | MIMAT0025857 | AL603750.1 | 4 | KAZALD1 |
| 11 | hsa-miR-335-5p | MIMAT0000765 | LUCAT1 | 13 | KAZALD1 |
| 21 | hsa-miR-335-5p | MIMAT0000765 | LINC01503 | 11 | KAZALD1 |
| 31 | hsa-miR-335-5p | MIMAT0000765 | AL391422.4 | 10 | KAZALD1 |
| 41 | hsa-miR-335-5p | MIMAT0000765 | IL10RB-DT | 9 | KAZALD1 |
| 51 | hsa-miR-335-5p | MIMAT0000765 | IL10RB-DT | 9 | KAZALD1 |
| 12 | hsa-miR-579-3p | MIMAT0003244 | OIP5-AS1 | 9 | RPRD1B |
| 22 | hsa-miR-579-3p | MIMAT0003244 | MAGI2-AS3 | 8 | RPRD1B |
| 32 | hsa-miR-579-3p | MIMAT0003244 | CARMN | 6 | RPRD1B |
| 42 | hsa-miR-579-3p | MIMAT0003244 | AC107027.3 | 6 | RPRD1B |
| 52 | hsa-miR-579-3p | MIMAT0003244 | AC107027.3 | 6 | RPRD1B |
| 13 | hsa-miR-664b-3p | MIMAT0022272 | SNHG16 | 13 | RPRD1B |
| 23 | hsa-miR-664b-3p | MIMAT0022272 | SNHG29 | 11 | RPRD1B |
| 33 | hsa-miR-664b-3p | MIMAT0022272 | LINC01521 | 11 | RPRD1B |
| 43 | hsa-miR-664b-3p | MIMAT0022272 | AL049555.1 | 11 | RPRD1B |
| 53 | hsa-miR-664b-3p | MIMAT0022272 | LINC00941 | 11 | RPRD1B |
| 14 | hsa-miR-1277-5p | MIMAT0022724 | SNHG14 | 4 | RPRD1B |
| 24 | hsa-miR-1277-5p | MIMAT0022724 | SNHG14 | 4 | RPRD1B |
| 34 | hsa-miR-1277-5p | MIMAT0022724 | SNHG14 | 4 | RPRD1B |
| 44 | hsa-miR-1277-5p | MIMAT0022724 | SNHG14 | 4 | RPRD1B |
| 54 | hsa-miR-1277-5p | MIMAT0022724 | SNHG14 | 4 | RPRD1B |
| 15 | hsa-miR-218-5p | MIMAT0000275 | SNHG16 | 12 | GLCE |
| 25 | hsa-miR-218-5p | MIMAT0000275 | SNHG12 | 10 | GLCE |
| 35 | hsa-miR-218-5p | MIMAT0000275 | LINC02595 | 8 | GLCE |
| 45 | hsa-miR-218-5p | MIMAT0000275 | AL109615.3 | 8 | GLCE |
| 55 | hsa-miR-218-5p | MIMAT0000275 | NEAT1 | 7 | GLCE |
| 16 | hsa-miR-448 | MIMAT0001532 | IRF1-AS1 | 7 | GLCE |
| 26 | hsa-miR-448 | MIMAT0001532 | AC040977.1 | 6 | GLCE |
| 36 | hsa-miR-448 | MIMAT0001532 | AP002807.1 | 5 | GLCE |
| 46 | hsa-miR-448 | MIMAT0001532 | AC021016.1 | 5 | GLCE |
| 56 | hsa-miR-448 | MIMAT0001532 | SH3BP5-AS1 | 4 | GLCE |
